# Metabolic reprogramming via mitochondrial delivery for enhanced maturation of chemically induced cardiomyocyte‐like cells

**DOI:** 10.1002/mco2.70005

**Published:** 2024-11-28

**Authors:** Yena Nam, Yoonji Song, Seung Ju Seo, Ga Ryang Ko, Seung Hyun Lee, Eunju Cha, Su Min Kwak, Sumin Kim, Mikyung Shin, Yoonhee Jin, Jung Seung Lee

**Affiliations:** ^1^ Department of Physiology Graduate School of Medical Science Brain Korea 21 Project Yonsei University College of Medicine Seoul Republic of Korea; ^2^ Department of Biomedical Engineering Sungkyunkwan University (SKKU) Suwon Republic of Korea; ^3^ Department of Intelligent Precision Healthcare Convergence Sungkyunkwan University (SKKU) Suwon Republic of Korea; ^4^ Department of Medicine College of Medicine Yonsei University Graduate School Seoul Republic of Korea; ^5^ Center for Neuroscience Imaging Research Institute for Basic Science (IBS) Suwon Republic of Korea; ^6^ Department of MetaBioHealth Sungkyunkwan University (SKKU) Suwon Republic of Korea

**Keywords:** cardiomyocytes, cell reprogramming, metabolic regulation, mitochondrial transfer

## Abstract

Heart degenerative diseases pose a significant challenge due to the limited ability of native heart to restore lost cardiomyocytes. Direct cellular reprogramming technology, particularly the use of small molecules, has emerged as a promising solution to prepare functional cardiomyocyte through faster and safer processes without genetic modification. However, current methods of direct reprogramming often exhibit low conversion efficiencies and immature characteristics of the generated cardiomyocytes, limiting their use in regenerative medicine. This study proposes the use of mitochondrial delivery to metabolically reprogram chemically induced cardiomyocyte‐like cells (CiCMs), fostering enhanced maturity and functionality. Our findings show that mitochondria sourced from high‐energy‐demand organs (liver, brain, and heart) can enhance structural maturation and metabolic functions. Notably, heart‐derived mitochondria resulted in CiCMs with a higher oxygen consumption rate capacity, enhanced electrical functionality, and higher sensitivity to hypoxic condition. These results are related to metabolic changes caused by increased number and size of mitochondria and activated mitochondrial fusion after mitochondrial treatment. In conclusion, our study suggests that mitochondrial delivery into CiCMs can be an effective strategy to promote cellular maturation, potentially contributing to the advancement of regenerative medicine and disease modeling.

## Graphical abstract

**Proposed mechanistic model of mitochondrial delivery inducing metabolic reprograming and maturation of CiCMs**. During the process of direct reprogramming, mitochondrial delivery modulates bioenergetics of immature CMs inducing maturation of the cells. After the treatment of functional mitochondria with robust OXPHOS activity, recipient mitochondria are replaced to mature status, and in turn induces CiCMs metabolically active. As a result, the mitochondria‐treated CiCMs can exhibit enhanced energy production, respiratory activity, electrophysiological functionality with organized intracellular structures and cardiac‐specific characteristics, resulting in mature CMs.

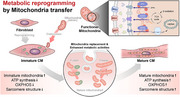

## 1 INTRODUCTION

Heart degeneration, following myocardial infarction or heart failure, constitute leading causes of global mortality.^1^, ^2^, ^3^ The limited ability of the myocardium to restore cardiomyocyte loss after injury has posed a significant challenge in regenerating the damaged heart. Direct cellular reprogramming technology offers a promising strategy for generating functional cardiomyocytes, providing a potential solution to this challenge.^4^ By employing forced expression of transcription factors (TFs) and/or microRNAs, somatic cells can be directly reprogrammed into cardiomyocytes both in vitro and in vivo.^5^ This method has shown encouraging results in functional recovery of the infarcted heart.^6^ While viral vectors have been effective in cardiac reprogramming through *TF* gene delivery, their clinical application is limited due to safety concerns.^7^ Recently, the use of small molecules has emerged as an alternative approach to induce direct reprogramming of primary mouse embryonic fibroblasts (PMEFs) into cardiomyocyte‐like cells (CiCMs),^2^, ^8^, ^9^ offering advantages such as lower immunogenicity and tumorigenicity compared to viral methods, while also often being faster and reversible.^10^, ^11^ Yet, a significant limitation emerges in the form of CiCMs generated through chemical reprogramming exhibiting low conversion efficiencies and fetal‐like cardiomyocyte characteristics.^12^ This immaturity of CiCMs hinders their applications in regenerative medicine from disease modeling to translational uses. To address the immaturity of CiCMs, several strategies have been proposed to enhance their maturation. These strategies include physical manipulation of cell substrates^13^ and three‐dimensional (3D) culture of the cells in extracellular matrices to provide in vivo‐like biochemical and biophysical cues.^14^, ^15^, ^16^, ^17^ However, they lack thorough exploration of metabolic maturation of CiCMs, which is pivotal in driving cardiomyocyte development and function.

Mitochondria are the key cell organelles playing pivotal roles in bioenergetics and physiological homeostasis. In most eukaryotic cells, mitochondria are in charge of adenosine triphosphate (ATP) production, biosynthesis of various biomolecules and calcium metabolism, which then regulate diverse cellular processes such as differentiation, proliferation, and even cell fate.^18^, ^19^, ^20^ Especially, these organelles are integral in cardiac development, function, disease, and profoundly influence cardiomyocyte maturation.^21^ The contractile function of cardiomyocytes heavily relies on energy availability, necessitating the significant increase in mitochondrial content through biogenesis as the fetal heart matures to the adult phenotype.^22^ Indeed, peroxisome proliferator‐activated receptor‐γ coactivator‐1 (PGC‐1), the main regulator of mitochondrial biogenesis, is known to increase in mouse heart directly after birth.^23^ Moreover, as cardiomyocytes mature, the primary energy pathway transitions from glycolysis into mitochondrial oxidative metabolism, using fatty acid as a main substrate,^21^, ^22^, ^24^, ^25^ which proposes metabolic reprogramming of CiCMs through mitochondrial transfer as a potential strategy to enhance cardiac maturation.

Mitochondrial transfer can rewire the metabolism of recipient cells, and eventually the cell fate, by driving changes in mitochondrial biogenesis, dynamics, and bioenergetic pathway. Generally, spontaneous mitochondrial transfer occurs between cells to maintain tissue homeostasis and development. Under stressful conditions, such as ischemia‐hypoxia, chemical exposure, or damages in mitochondrial DNA (mtDNA), intercellular mitochondrial transfer appears to rescue tissue damage and revitalize exhausted cells by replacing damaged mitochondria with insufficient energy supply.^26^ In the same context, artificial mitochondrial transfer supplies exogenous mitochondria to promote cellular metabolism in a simple and quick way. Mitochondrial transplantation induces increase in mitochondrial biogenesis and improves mitochondrial oxidative respiration, which is critical in energy supply for cellular reprogramming or curative responses.^27^ Previous study has shown that mitochondrial transfer from mesenchymal stem cells could reprogram adult cardiomyocytes toward a progenitor‐like state.^28^ Another study showed that mitochondrial transfer from iPSC‐CMs restored myocardial bioenergetics in ischemic myocardium in vivo.^29^ These findings suggest that cellular transfer of mitochondria could play a role in altering cell fate and tissue repair reprogramming via energetic modulation. Given this, we hypothesized that mitochondria sourced from high‐energy‐demand organs might enhance energetics and force generation of CiCMs, crucial for the utility of CiCMs in cardiac regeneration as well as drug screening applications.

Mitochondria were extracted from the heart, brain, and liver tissues, which are recognized for their higher energy requirements and robust respiratory capacity compared to other tissue types.^30^, ^31^, ^32^ Subsequently, they were introduced to cells during the process of reprogramming for enhancing the maturity of CiCMs. Our comparison revealed that CiCMs with transplanted mitochondria displayed more mature phenotypes, as evidenced by the organization of sarcomeres, intercellular calcium transients, and metabolic functions, in contrast to CiCMs without transferred mitochondria. Further, CiCMs that received mitochondria from heart tissue exhibited higher oxygen consumption rate capacity and showed marginally superior mature phenotypes than those which received liver and brain mitochondria. Collectively, we report a novel and potent strategy for generating functional CiCMs through metabolic reprogramming of cells by the transplantation of mitochondria.

## 2 RESULTS

### 2.1 Mitochondrial characteristics and functional analysis across different tissues

**FIGURE 1:**
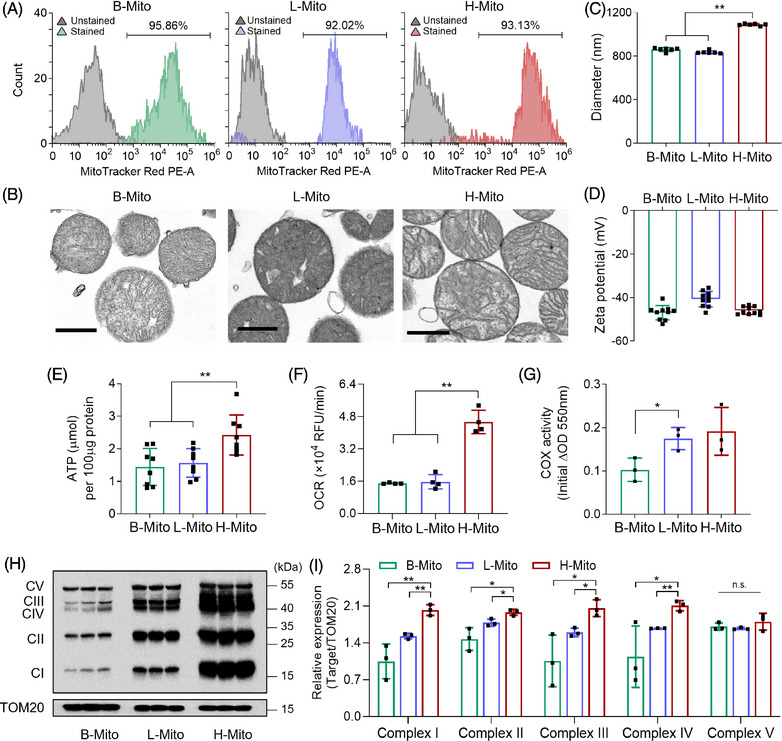
Mitochondrial characteristics and functional analysis across different tissues. (A) Flow cytometry analysis using MitoTracker Red to verify mitochondrial purity. (B) Transmission electron microscopy (TEM) images of B‐Mito, L‐Mito, and H‐Mito (scale bars = 500 nm). (C) Size (*n* = 6) and (D) zeta potential (*n* = 10) of isolated mitochondria. (E) ATP levels of the freshly isolated mitochondria (*n* = 8). (F) Oxidative phosphorylation (OXPHOS) activities of isolated mitochondria determined by extracellular oxygen consumption rate (OCR) assay (*n* = 4). (G) Cytochrome *c* oxidase activity reflecting an initial burst of cytochrome *c* oxidation (*n* = 3). (H) Western blot analysis of mitochondrial OXPHOS complex protein subunits and TOM20 as a loading control. Complex I, NDUFB8 (20 kDa); Complex II, SDHB (30 kDa); Complex III, UQCRC2 (48 kDa); Complex IV, MTCO1 (40 kDa); Complex V, ATP5A (55 kDa). (I) Relative protein expression of mitochondria isolated from different tissues quantified by densitometry and normalized to that of TOM20 (*n* = 3). All data are expressed as the means ± SD. Statistical difference between the groups was determined by two‐tailed *t*‐test (*p < 0.05 and **p < 0.01).

To optimize acquisition and source of mitochondria for metabolic reprogramming, we isolated mitochondria from different tissue sources, brain (B‐Mito), liver (L‐Mito) and heart (H‐Mito), using a series of homogenization and centrifugations. Generally, these three organs are known for high energy demand and robust mitochondrial activities in mammals,^30^, ^31^, ^32^ and have been commonly used as mitochondrial donor sources due to their metabolic profiles, organelle density, and ease of access to organelles.^33^, ^34^, ^35^, ^36^ The purity of isolated mitochondria was confirmed using MitoTracker Red which stains active mitochondria,^37^ followed by flow cytometry analysis. Approximately 95.86 ± 1.7 %, 92.02 ± 1.3 %, and 93.13 ± 1.0 % of the mitochondria from brain, liver, and heart, respectively, were active (Figure 1A). Moreover, electron microscopy revealed well‐preserved mitochondrial ultrastructures, including inner membrane and matrix structures without notable deformation after the isolation (Figure 1B). The mean sizes of isolated mitochondria were 860 ± 17 nm (B‐Mito), 835 ± 13 nm (L‐Mito), and 1090 ± 9 nm (H‐Mito) (Figure 1C), which are in the commonly known diameter range of mitochondria (0.5 ≈ 2 𝜇m) under isotonic conditions.^38^ Interestingly, H‐Mito displayed the largest mean size among the tissues, appearing to be a bioenergetically optimal strategy for its high energy requirement to facilitate continuous contraction and relaxation cycles.^39^ Mitochondrial surface charges, determined by dynamic light scattering, exhibited negative charges in all three groups (‐46.97 ± 3.28 mV for B‐Mito, ‐40.7 ± 3.57 mV for L‐Mito, and ‐45.99 ± 1.75 mV for H‐Mito), likely due to their highly negatively charged matrix (Figure 1D).^40^, ^41^

Next, mitochondria from different tissue sources were compared in terms of bioenergetic functions to maximize metabolic reprogramming efficiency after transferring the mitochondria into CiCMs (Figure 1E–G). We first measured the mitochondrial ATP from the same concentration of purified mitochondria, and H‐Mito exhibited the highest level of ATP production (Figure 1E). The energy capacity of mitochondria was further tested by examining oxidative phosphorylation (OXPHOS), a chemical energy releasing pathway to synthesize ATP. Since oxygen is the final acceptor in the electron transport chain during mitochondrial aerobic respiration, the oxygen consumption rate (OCR) provides important information about OXPHOS function.^42^ The result indicated that H‐Mito exhibits significantly higher respiratory capacity than B‐Mito and L‐Mito (Figure 1F). In addition, cytochrome *c* oxidase (COX) activity assay was performed across organs because COX is the enzyme constituting the last step of the respiratory chain, and its activity can be used as the reference for the activity of OXPHOS system.^43^ The obtained result (Figure 1G) indicated that H‐Mito demonstrated the most vigorous enzymatic activity, which is consistent with the previous finding.^43^ The expression of mitochondrial proteins associated with OXPHOS pathway was also evaluated, and all the OXPHOS complex subunits (I, II, III, IV, and V) were remarkably overexpressed in H‐Mito, compared to mitochondria isolated from brain and liver (Figures 1H,I and S1). Taken together, these data suggest that H‐Mito has superior respiratory capacity than B‐Mito and L‐Mito, making it as the most suitable mitochondrial donor source.

### 2.2 Delivery of the mitochondria isolated from the adult tissues into CiCMs

**FIGURE 2:**
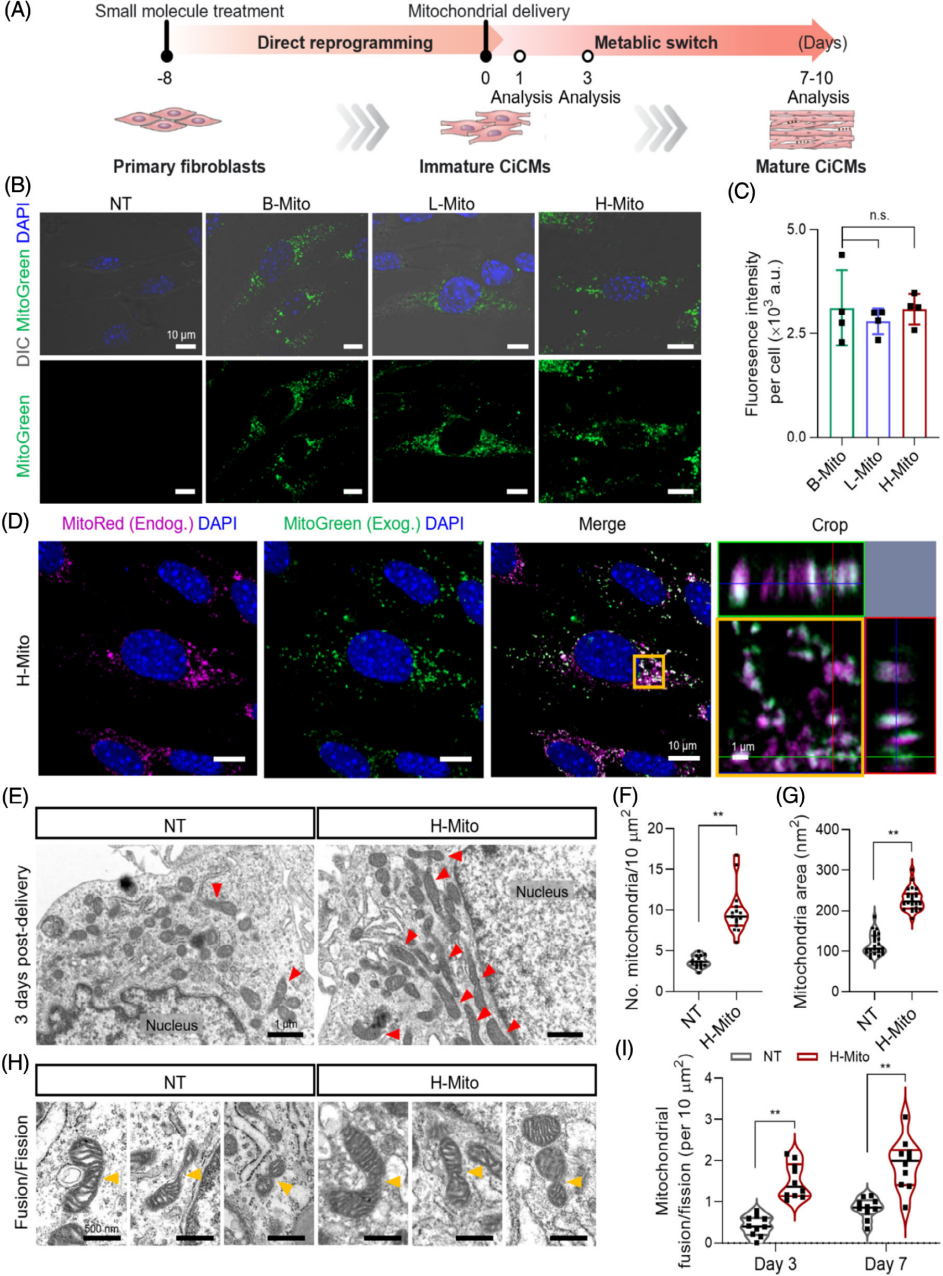
Uptake of tissue‐derived mitochondria by chemically induced cardiomyocyte‐like cells (CiCMs). (A) Schematic timeline of generating induced CiCMs by chemical induction and mitochondrial delivery isolated from adult tissues. (B) Fluorescent images illustrating CiCMs during chemical cardiac reprogramming, 24 h post‐transfection with MitoTracker Green‐labeled mitochondria isolated from various adult tissues (scale bars = 10 µm). (C) Analysis of MitoTracker Green fluorescence intensity per cell, representing internalized mitochondria (*n* = 4). (D) Images showing co‐localization of exogenous mitochondria (Exog.), labeled with MitoTracker Green, and the cell's endogenous mitochondria (Endog.), marked with MitoTracker Red. A higher magnification image (right) illustrates the coexistence of transfected and native mitochondria (scale bars = 10 µm for the main image, and 1 µm for the magnified image). (E) Transmission electron microscopy (TEM) images of CiCMs after 3 days of transfection with H‐Mito (scale bars = 1 µm). Elongated mitochondria are marked with red arrows. Quantitative analysis of (F) mitochondrial count per µm^2^ (*n* = 15), and (G) average mitochondrial size in terms of area (*n* = 24). (H) Representative TEM images of CiCMs at 3 days post‐transfection with H‐Mito, depicting mitochondrial fusion/fission events (indicated by yellow arrows, scale bars = 500 nm), and (I) quantitative analysis of these mitochondrial dynamics (*n* = 10 at each time point). All data are expressed as the means ± SD. Statistical difference between the groups was determined by (C, F, G) two‐tailed *t*‐test or (I) Two‐way ANOVA followed by Bonferroni correction (*p < 0.05 and **p < 0.01 versus NT group).

We introduced mitochondria isolated from brain, liver, and heart tissues into cells undergoing cardiac reprogramming on day 8 of postchemical induction (Figure 2A). For tracking the incorporation of mitochondria during coculture with CiCMs, the isolated mitochondria were labeled using MitoTracker Green, and the successful delivery of mitochondria into CiCMs after 24 h was demonstrated across all groups (B‐Mito, L‐Mito, H‐Mito), with each exhibiting a similar fluorescence intensity per cell (Figure 2B,C). This implies that the efficiency of mitochondrial delivery is independent of the tissue origin. To further validate delivery of the treated mitochondria, we additionally conducted flow cytometry analysis of CiCMs after treating B‐Mito, L‐Mito, or H‐Mito. After 24 h of mitochondrial treatment, over 98% of CiCMs in all groups (B‐Mito 98.21 ± 0.32%; L‐Mito 98.54 ± 0.24%; H‐Mito 98.93 ± 0.09%) expressed fluorescent signal, demonstrating successful transfer of the mitochondria into CiCMs with no significant difference of delivery efficiency by tissue origin (Figure S2A). Furthermore, we performed qPCR to quantify the mtDNA in CiCMs after the delivery of exogenous mitochondria. The relative mtDNA copy numbers in B‐Mito, L‐Mito, and H‐Mito groups compared to non‐treated (NT) CiCMs were 1.76   ± 0.03, 1.75   ± 0.03, and 1.86 ± 0.06, respectively, after 24 hours of delivery, indicating that similar amount of mitochondria were delivered into CiCMs (Figure S2B). Combining these results, we could conclude that the delivery efficiency and the amount of delivered mitochondria into CiCMs were similar regardless of tissue origin, and the regulation of CiCMs maturation might be solely affected by the intrinsic activity of mitochondria. Next, dynamic interaction between the delivered mitochondria and the mitochondria in recipient cells was observed after the treatment (Figure 2D). Following a previous study, mitochondria are known to be integrated into cells by endocytosis and induce changes in mitochondrial network through fusion with the mitochondria in the host cells, leading to metabolic reprogramming.^44^ When stained with fluorescent dyes (green; exogenous mitochondria, magenta; endogenous mitochondria), H‐Mito exhibited overlap with MitoTracker Red‐stained recipient mitochondria at 24 h, indicating effective mitochondrial transfer and the occurrence of mitochondrial fusion events (Figure 2D). To further validate the mitochondrial fusion/fission events in a protein level, we performed western blot analysis of mitochondrial dynamics protein including mitofusin 2 (MFN2), optic atrophy 1 (OPA1) and dynamin‐related protein 1 (DRP1) 24 h post heart mitochondrial delivery. MFN2 and OPA1 are key regulators of mitochondrial fusion, located in the mitochondrial outer membrane and inner membrane, respectively. Conversely, DRP1 is in charge of mitochondrial fission by interacting with receptor proteins such as fission 1 protein (FIS1), mitochondrial fission factor (MFF), and mitochondrial dynamics proteins of 49 and 51 kDa (MID49/51) located at mitochondrial outer membrane.^45^ As a result, expression of mitochondrial fission protein, DRP1, slightly increased in the H‐Mito group, but the difference from the NT group was not significant (Figures S3A, S3B and S4). However, mitochondrial fusion protein MFN2 and OPA1 expression greatly increased when H‐Mito was treated, implying enhanced mitochondrial fusion activity after mitochondrial treatment (Figures S3A, S3B and S4). Previous studies also reported that the upregulation of MFN2 and OPA1 is in connection with increased mitochondrial fusion and elongation.^46^, ^47^, ^48^ In addition, we also have observed that OXPHOS protein subunits I to V expressions significantly increased in H‐Mito group after 24 h post mitochondrial delivery, providing potential for metabolic enhancement by H‐Mito internalization (Figures S3C and S4). In addition, transmission electron microscopy (TEM) was also employed to evaluate the changes of mitochondria within the recipient cells following mitochondrial delivery (Figures 2E–I and S5). The analysis revealed that the CiCMs treated with H‐Mito not only exhibited an increased average number of mitochondria (Figure 2E,F) but also a substantially larger mitochondrial size compared to the CiCMs with no treatment (NT) (Figures 2E,G,H and S5). Together with increased mitochondrial fusion, mitochondrial morphology in H‐Mito group showed more elongated and tubular network with dense cristae, enabling robust respiration (Figures 2E,H and S5).^49^ Importantly, a higher number of mitochondria were observed to be undergoing fusion or fission after both 3 and 7 days of mitochondrial delivery (Figure 2H,I). These results suggest that the introduction of H‐Mito leads to enhanced mitochondrial biogenesis and promotes a shift toward increased fusion/fission events within CiCMs.

### 2.3 Delivery of mitochondria promotes CiCM maturation

**FIGURE 3:**
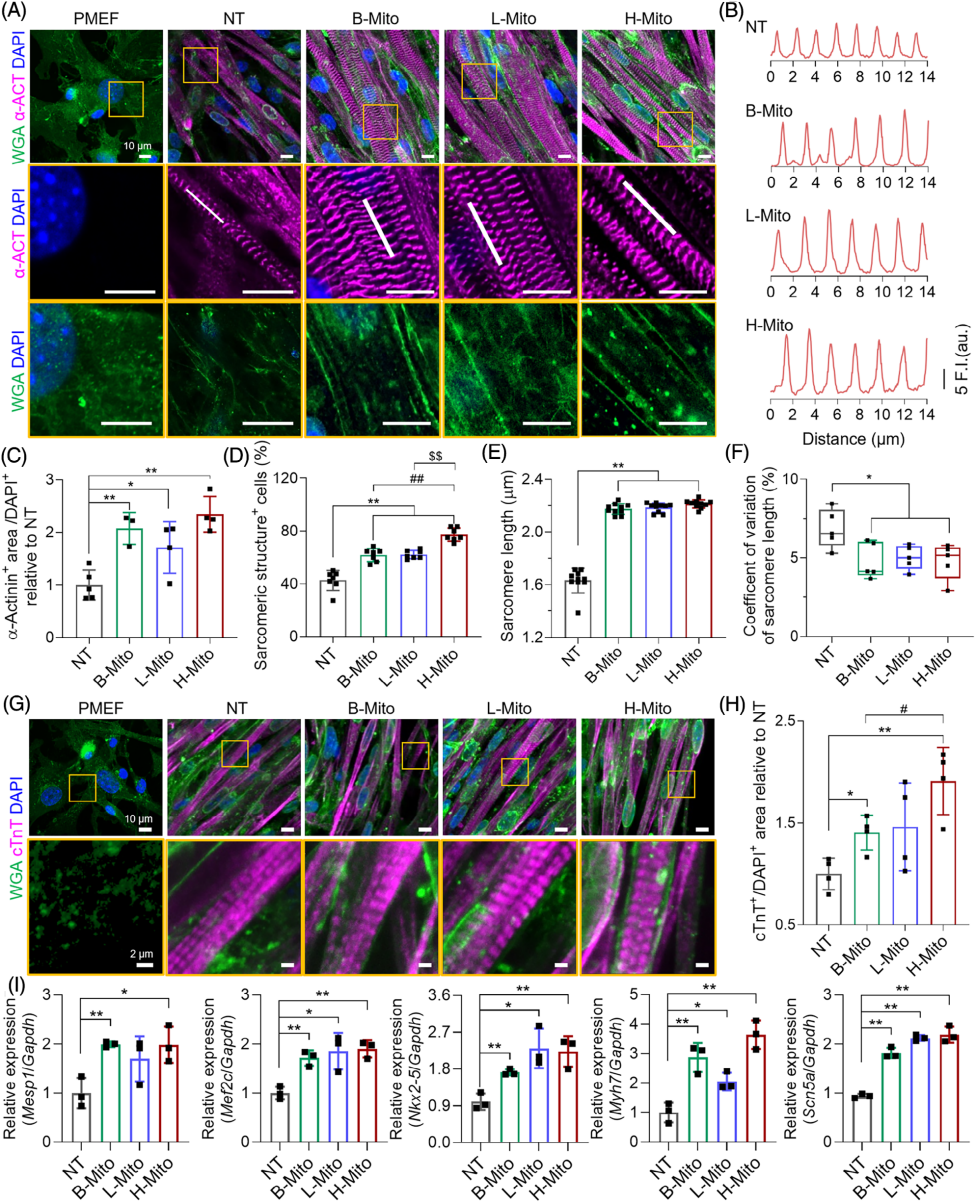
Enhanced maturation of chemically induced cardiomyocyte‐like cells (CiCMs) induced by mitochondrial treatment. (A) Immunostaining for alpha‐actinin (α‐ACT) and t‐tubules (detected by FITC‐conjugated WGA) in PMEFs and CiCMs, both untreated (No Treat; NT) and those treated with mitochondria derived from brain, liver, and heart tissues (B‐Mito, L‐Mito, H‐Mito) for 7 days (scale bars = 10 µm). (B) Sarcomere length (SL) analysis derived from the region of interest on α‐ACT immunostained images (indicated by a white line in the second‐row images). Quantifications of (C) α‐ACT+ area (*n* = 4), (D) cells with sarcomere structures (*n* = 7), (E) average SL (*n* = 10), and (F) coefficient of variation of SL (*n* = 5) based on α‐ACT immunostained images. (G) Immunostaining for cardiac troponin T (cTnT) and WGA in PMEF, B‐Mito, L‐Mito, and H‐Mito groups after 7 days of mitochondrial treatment (corresponding to 15 days of CiCM culture) (scale bars = 10 and 2 µm for magnified images). (H) Quantification of cTnT‐positive area (*n* = 4). (I) Quantitative reverse transcription polymerase chain reaction (qRT‐PCR) analysis of cardiac‐specific genes in various CiCM groups (*n* = 3). All data are expressed as the means ± SD. Statistical difference between the groups was determined by two‐tailed *t*‐test (*p < 0.05 and **p < 0.01 versus NT, #*p* < 0.05 and ##*p* < 0.01 versus B‐Mito, and $$*p* < 0.01 versus L‐Mito group).

The efficiency of direct cardiac reprogramming in each condition (NT, B‐Mito, L‐Mito, and H‐Mito) was investigated by analyzing the expression of representative cardiac proteins [α‐actinin and cardiac troponin‐T (cTnT)] (Figure 3A–H). On day 7 of mitochondrial delivery, α‐actinin positive cells with clear cross‐striated patterns started to appear in all CiCMs (Figure 3A,B). Individual CiCMs in mitochondria‐treated groups also displayed a long, rod‐like shape, which is typical in mature cardiomyocytes (Figure S6). Especially, mitochondria‐treated groups (B‐Mito, L‐Mito, and H‐Mito) exhibited significantly higher percentages of *α*‐actinin positive cells (Figure 3C) and sarcomere structure containing cells (Figure 3D) with longer average sarcomere lengths (Figure 3E). In addition, there was reduced variation of sarcomere length in mitochondria‐delivered groups compared to NT group (Figure 3F). We also observed a more frequent development of transverse tubules (t‐tubules) in the CiCMs treated with B‐Mito, L‐Mito, and H‐Mito, as confirmed by wheat germ agglutinin labeling (WGA; Figure 3A). Similarly, cTnT positive cells with clear cross‐striated patterns started to appear at day 7 in B‐Mito, L‐Mito, and H‐Mito treated groups, with H‐Mito showing the largest cTnT expressing area (Figure 3G,H). These results indicate that mitochondrial delivery might enhance the development and maturation of sarcomere structures within CiCMs.

Changes of cardiac specific markers after mitochondrial delivery were further investigated by using quantitative real‐time polymerase chain reaction (qRT‐PCR) analysis. After 7 days of mitochondrial treatment, there were substantial increases in cardiac gene (*Mesp1, Mef2c*, *Nkx2‐5, Myh7*and *Scn5a*) expression in all mitochondria‐treated groups compared with untreated control (Figure 3I). In specific, mitochondrial delivery significantly enhanced expression of cardiac progenitor markers (*Mesp1, Mef2c*, *Nkx2‐5*). The expression of *Myh7*, one of the early cardiomyocyte markers, was even about 3 times higher in B‐Mito and H‐Mito groups compared to NT group. In addition, the level of *Scn5a*, a mature cardiomyocyte marker, was also about two times greater in the mitochondria‐treated groups than NT group, which demonstrates enhanced cardiac differentiation and maturation by mitochondrial treatment. Especially, H‐Mito group exhibited the most significant upregulation in all tested cardiac genes, implying the highest reprogramming efficiency.

### 2.4 Metabolically reprogrammed CiCMs are structurally and bioenergetically mature

**FIGURE 4:**
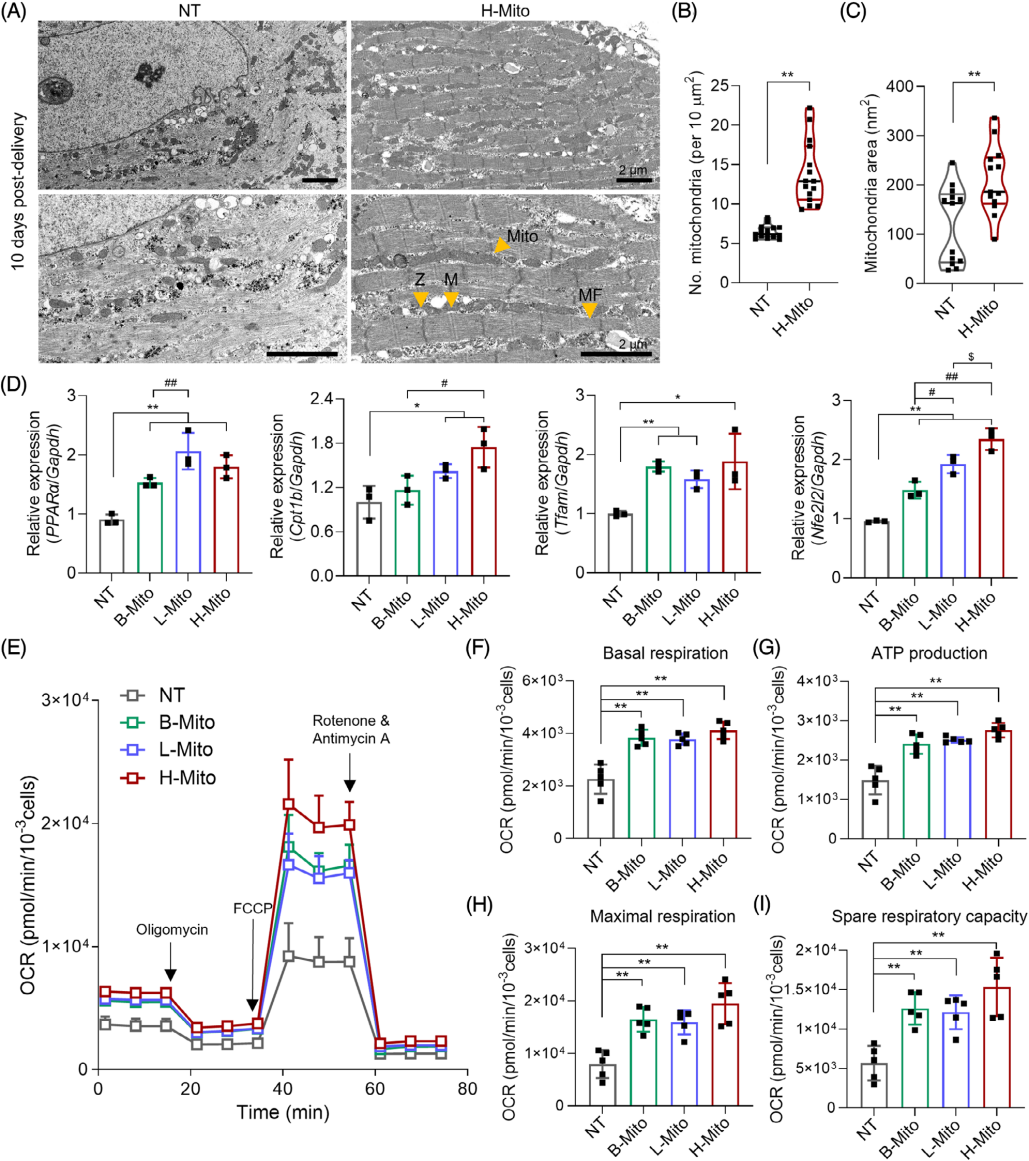
Mitochondrial reprogramming enhances ultrastructure and bioenergetics in chemically induced cardiomyocyte‐like cells (CiCMs). (A) Transmission electron microscopy (TEM) images of CiCMs after 10 days of treatment with H‐Mito (Annotations: mito = mitochondria, Z = Z‐line, M = M‐line, MF = myofibril, scale bars = 2 µm). Quantitative analysis of (B) mitochondrial count per 10 µm^2^ (*n* = 15), and (C) average mitochondrial size in terms of area (*n* = 13). (D) Quantitative reverse transcription polymerase chain reaction (qRT‐PCR) analysis of metabolism‐related genes in various CiCM groups (*n* = 3). (E) Oxygen consumption rate (OCR) measurements in untreated CiCMs (NT), and those treated with mitochondria from brain, liver, and heart tissues (*n* = 5). Basal respiration was observed for 72 min. Oligomycin, which inhibits ATP production via oxidative phosphorylation, was introduced at 16 min. Subsequently, carbonyl cyanide p‐trifluoro methoxyphenylhydrazone (FCCP) was injected at 36 min, followed by complex I and III inhibitors, rotenone and antimycin A, respectively. The quantitative analysis includes (F) basal respiration (*n* = 5), (G) ATP production (*n* = 5), (H) maximal respiration (*n* = 5), and (I) spare respiratory capacity (*n* = 5). Statistical significance between groups was determined using a two‐tailed t‐test (*p < 0.05 and **p < 0.01 versus NT, #*p* < 0.05 and ##*p* < 0.01 versus B‐Mito, and $*P* < 0.05 versus L‐Mito group).

Given the essential role of mitochondria in cellular energy supply and subsequent energetic modulation, cytoplasmic alteration in CiCMs was investigated using TEM. Ultrastructural analysis conducted after 10 days of H‐Mito treatment revealed enhanced myofibrillar organization in CiCMs, indicative of more advanced morphological development (Figure 4A–C). H‐Mito delivery induced substantial improvements in the arrangement and density of myofibrils, with Z‐lines and M‐lines, and an array of elongated mitochondria. In contrast, the NT group displayed an immature contractile apparatus, with sparse and misaligned myofibrils (Figure 4A). Additionally, the H‐Mito group exhibited a statistically higher mitochondrial count (Figure 4B) and an increase in average mitochondrial size compared to the NT group (Figure 4C).

Expression of metabolic genes (*PPARα*, *Cpt1b*, *Tfam* and *Nfe2l2*) were also evaluated using qPCR (Figure 4D). Peroxisome proliferator activated receptor‐*α* (PPAR*α*) is highly expressed in tissues with a high capacity for mitochondrial fatty acid oxidation and known to regulate energy and lipid homeostasis.^50^, ^51^, ^52^, ^53^ Carnitine palmitoyltransferase‐1b (Cpt1b) is a rate‐determining enzyme of mitochondrial *β*‐oxidation, governing the mitochondrial entry of long‐chain fatty acid.^54^, ^55^, ^56^ Mitochondrial transcription factor A (Tfam) is essential for maintenance of mitochondrial DNA and for cell survival.^57^, ^58^, ^59^, ^60^ Lastly, nuclear factor erythroid 2‐related factor 2 (Nfe2l2) is a master regulator of cellular redox homeostasis that affects mitochondrial membrane potential, ATP synthesis, fatty acid oxidation, and mitochondrial structural and functional integrity.^61^ The expression of these genes, referring metabolic activities, was enhanced in all mitochondria‐receiving groups compared to the NT group, with H‐Mito showcasing the highest expression for all (Figure 4D), which also supports correlation between metabolic enhancement and maturation of CiCMs.

Mitochondria, which account for nearly 95% of cellular ATP production through OXPHOS, are vital for maintaining energy supply. Therefore, the rate of OXPHOS, represented by the OCR was measured after 7 days of mitochondrial treatment. As a result, mitochondrial treatment regardless of tissue origin resulted in a marked increase in OCR kinetics of CiCMs compared to the NT group (Figure 4E). In specific, a statistically significant augmentation in basal respiration (Figure 4F) and ATP production (Figure 4G) was recorded in the mitochondria‐treated groups, indicating enhanced metabolic activity. Furthermore, maximal respiration (Figure 4H) and the spare respiratory capacity (Figure 4I), which reflects a cellular ability to respond to an increase in energy demand or acute/chronic stress, were also notably enhanced when mitochondria were treated to CiCMs. To verify the metabolic reprogramming observed in the mitochondrial‐treated groups, we additionally performed a glycolytic rate assay (Figure S7). Our results show that basal glycolysis was significantly increased in CiCMs treated with B‐Mito and H‐Mito compared to untreated CiCMs (NT). The calculation of the OCR to extracellular acidification rate (ECAR) ratio revealed that the B‐Mito and H‐Mito groups exhibited a higher ratio compared to the NT, and L‐Mito groups, suggesting that the CiCMs treated with B‐Mito or H‐Mito rely more on oxidative metabolism than the glycolysis, despite the increased glycolytic activity. Together, the observed increase in OCR kinetics and OCR to ECAR ratio 7 days post‐delivery of mitochondria suggests an improvement in cellular bioenergetics and increased mitochondrial oxidative metabolism, likely attributable to the presence of mature mitochondrial content.

### 2.5 Mitochondrial delivery enhances electrical functionality in CiCMs

**FIGURE 5:**
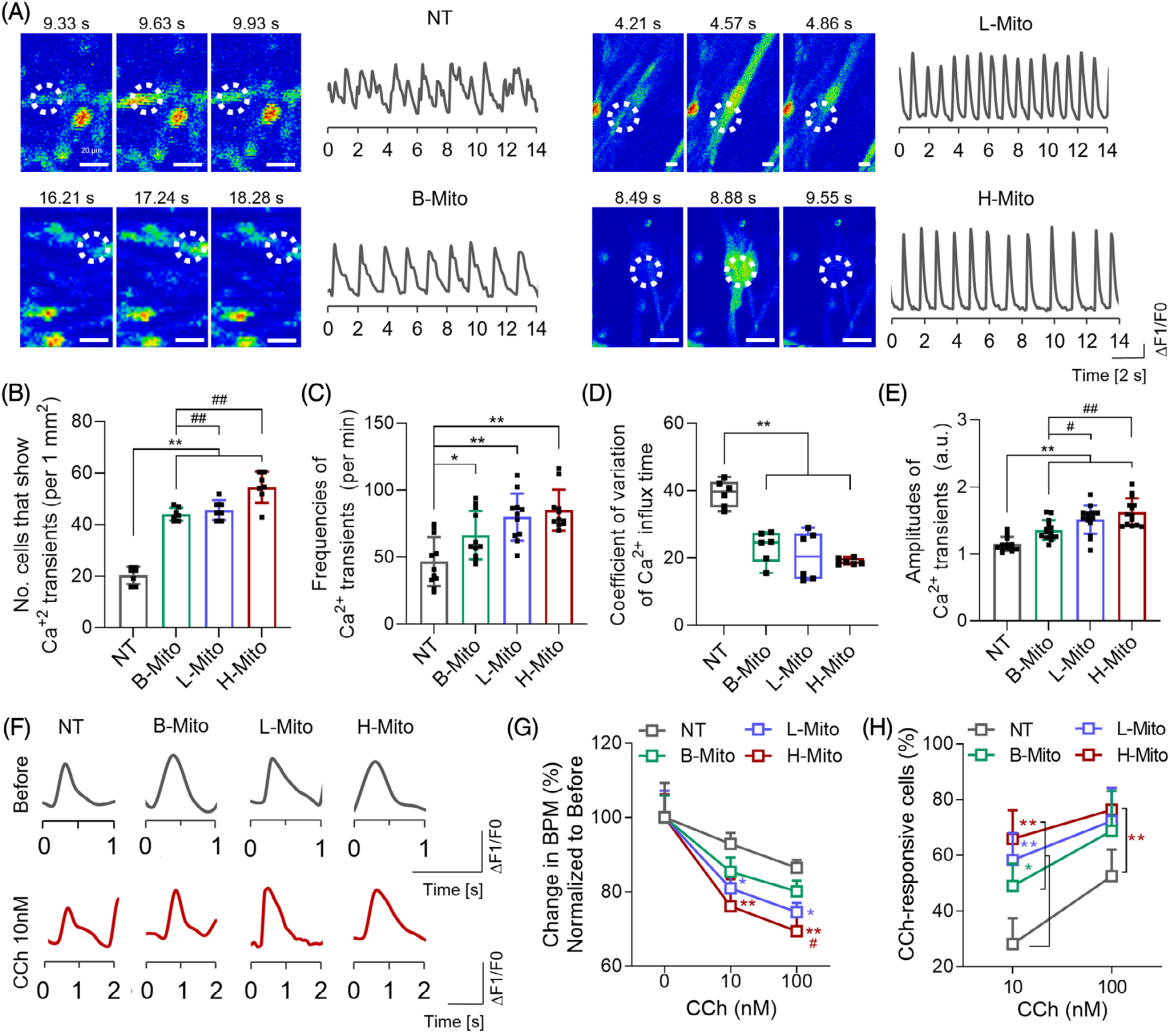
Calcium influx characterization of metabolically reprogrammed chemically induced cardiomyocyte‐like cells (CiCMs). (A) Representative time‐lapse calcium imaging in CiCMs loaded with the intracellular Ca^2+^ indicator Fluo‐4 AM (scale bars = 20 µm). The traces depicting changes in fluorescence intensity are shown in the area marked by a white dotted circle. (B) Average number of cells per square millimeter exhibiting spontaneous Ca^2+^ transients (*n* = 7). (C) Mean frequency of Ca^2+^ transients occurring per minute (*n* = 11). (D) Coefficient of variation for the duration of Ca^2+^ transients (*n* = 6). (E) Average amplitude of Ca^2+^ transients (*n* = 14). (F) Representative traces of Ca^2+^ fluorescence pre‐ and post‐treatment with 10 nM carbamylcholine (CCh). (G) Change in beating per minute (BMP) normalized to pre‐treatment of CCh (*n* = 4). (H) Quantification of CCh‐responsive cells, demonstrated by a reduction in beats per minute (BPM). Statistical significance between groups was determined using (B‐E) a two‐tailed *t*‐test or (G, H) two‐way ANOVA followed by Tukey (*p < 0.05 and **p < 0.01 versus NT, and #*p* < 0.05 and ##*p* < 0.01 versus B‐Mito group).

To more closely examine the differentiation and maturation of CiCMs among the population of contracting cells, we investigated electrophysiological functions of the cells by confirming calcium flux patterns and response to chronotropic agents 7 days following treatment with H‐Mito (Figure 5). Spontaneous calcium transients in CiCMs were examined by conducting a time‐lapse imaging analysis of calcium influx using Fluo‐4, a fluorescently labeled calcium indicator (Figure 5A–E). The average numbers of cells showing spontaneous calcium transients were significantly higher in the mitochondria‐treated groups compared to the NT group (Figure 5B), with H‐Mito group exhibiting the highest count of spontaneous calcium influx cells (Figure 5B). Additionally, the frequencies of Ca^2+^ transients were markedly increased in B‐Mito, L‐Mito, and H‐Mito groups relative to the NT group (Figure 5C). Mitochondria‐treated cells also displayed more regular calcium influxes with higher peak amplitudes, in contrast to the CiCMs in the NT group, which showed more irregular and the lower amplitudes of calcium transients (Figure 5A,D,E).

We then investigated the responsiveness of CiCMs to carbamylcholine (CCh), a muscarinic agonist known to decelerate cardiomyocyte beating rates (Figure 5F–H).^62^, ^63^, ^64^ Upon exposure to 10 nM and 100 nM CCh, CiCMs from all groups revealed reduced amplitudes of Ca^2+^ transients (Figure 5F,G). CiCMs across the board showed a decline in the frequency of Ca^2+^ transients (92.9% ± 1.5% for NT, 85.4% ± 1.9% for B‐Mito, 80.9 ± 1.5% for L‐Mito, and 76.1% ± 3.6% for H‐Mito at 10 nM CCh), underscoring a robust muscarinic signaling response (Figure 5G). The drop in beats per minute (BPM) following CCh exposure was more marked in the mitochondria‐treated groups than in the NT group, with H‐Mito exhibiting the most significant reduction at both CCh concentrations. This decrease in BPM was also observed to be dose‐dependent, with more pronounced reductions at higher CCh concentrations. Furthermore, the mitochondria‐treated groups exhibited significantly higher percentages of cells responsive to CCh, as evidenced by BPM following treatment with CCh, compared to the NT group (Figure 5H). These results demonstrate that CiCMs, especially treated with mitochondria derived from heart tissue, exhibited enhanced responsiveness to muscarinic signaling, which might suggest a superior maturation and functional status than the untreated CiCMs. These observations lend further support to the notion that mitochondrial delivery induces maturation and improves functionality of CiCMs.

### 2.6 Metabolic reprogramming increases sensitivity of CiCMs to hypoxia

**FIGURE 6:**
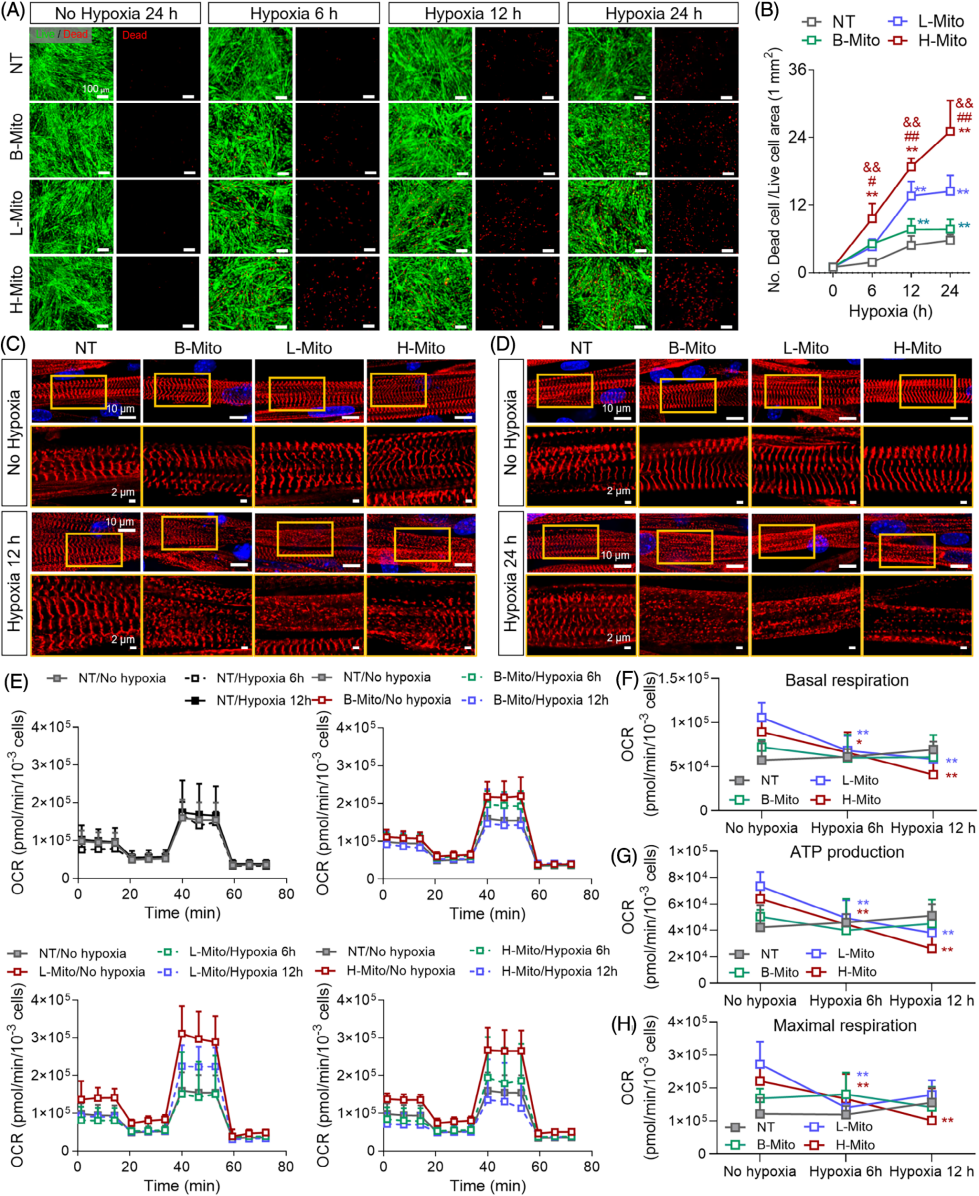
Metabolically matured chemically induced cardiomyocyte‐like cells (CiCMs) via mitochondrial delivery are more sensitive to hypoxia. (A) Live/dead assay by EthD‐1/calcein AM staining of CiCMs in NT, B‐Mito, L‐Mito, and H‐Mito groups exposed to 1% O_2_ for 6, 12, and 24 h (*n* = 4). (B) Quantitative analysis of the number of dead cells in terms of fluorescence area in different groups (*n *= 4). Immunostaining for alpha‐actinin (α‐ACT) in different CiCM groups exposed to 1% hypoxia for (C) 12 h, and (D) 24 h (scale bars = 10 and 2 µm for magnified images). (E) Real‐time oxygen consumption rate (OCR) of CiCMs in different groups exposed to 1% hypoxia for 6 and 12 h (*n* = 9). Cells were treated with oligomycin, carbonyl cyanide‐p‐trifluoromethoxyphenylhydrazone (FCCP), and antimycin A and rotenone to measure mitochondrial respiration. The quantitative analysis includes (F) basal respiration (*n* = 9), (G) ATP production (*n* = 9), and (H) maximal respiration (*n* = 9). Statistical significance between groups was determined using (B), (F), (G), and (H) two‐way ANOVA followed by Tukey **p < 0.01 versus NT, and #*p* < 0.05 and ##*p* < 0.01 versus B‐Mito group, and &&*p* < 0.01 versus L‐Mito group).

Finally, we have investigated whether metabolically reprogrammed CiCMs are more sensitive in hypoxia conditions (Figure 6).^65^ Previous studies suggest that metabolic maturation heightens cardiomyocyte susceptibility to hypoxia, resembling clinically relevant phenotype.^66^ Such mature cells exhibit significantly reduced mitochondrial respiration and increased cell death under prolonged hypoxic stress. To evaluate the hypoxic sensitivity of CiCMs, both with and without mitochondrial delivery, cells were subjected to 1% O_2_, and the cellular viability was determined using the Live/Dead assay after 6 h, 12 h, and 24 h (Figure 6A,B). The groups that received mitochondria exhibited reduced viability under hypoxic conditions when compared to the NT group. Notably, the H‐Mito group displayed the most significant reduction in survival rates at all time points, which further demonstrate the enhanced metabolic maturation and susceptibility to hypoxic condition (Figure 6B). Additionally, the impact of hypoxia on sarcomere structure was assessed by examining α‐actinin expression through immunostaining at 12 h and 24 h post‐hypoxia. There were no remarkable changes in the structure of sarcomere under normoxia in all groups until 24 h (Figure 6C,D). However, after hypoxic induction, CiCMs reprogrammed by mitochondrial delivery exhibited damaged and unclear striated patterns of sarcomere than those seen in the NT group, indicating increased susceptibility of the cells to hypoxia (Figure 6C,D). In addition, a comparison of the OCR between the NT group and the mitochondria‐treated groups was performed 6 h and 12 h after hypoxia induction (Figure 6E–H). Consistent to earlier study focusing on the bioenergetic enhancement of CiCMs after the mitochondrial delivery (Figure 4E–I), it was found that mitochondria‐treated groups demonstrated significant improvements in basal respiration, ATP production, and maximal respiration. However, after hypoxia induction, the mitochondria‐treated groups, with the exception of the B‐Mito group, displayed significant decrease in basal respiration (Figure 6F), ATP production (Figure 6G), and maximal respiration rates (Figure 6H). Consequently, these significant changes in OCR kinetics post‐hypoxia together with the structural damage indicate that the mitochondria‐treated groups, especially the H‐Mito group, are more adversely affected by hypoxic conditions.

## 3 DISCUSSIONS

Throughout the study, we have proposed metabolic reprogramming via mitochondrial transfer as a promising strategy to enhance maturation of CiCMs. Current methods to provide CiCMs are rather safe and fast, but there is a significant limitation of maturation, resulting in the generation of cells with fetal‐like cardiomyocyte characteristics. At the same time, it is well known that major metabolic pathways of cardiomyocytes change from glucose metabolism to fatty acid oxidation as they mature, along with morphological and physiological changes in endogenous mitochondrial network.^21^, ^22^, ^24^, ^25^ Also, bioenergetics are critical to support specialized functions, such as contraction, in adult cardiomyocytes. Previous studies have suggested that mitochondrial transfer can enhance mitochondrial oxidative metabolism by modulating biogenesis and dynamics of mitochondria.^67^, ^68^ Thus, we hypothesized that mitochondria sourced from bioenergetic tissues can enhance maturation of CiCMs by metabolically reprogramming cells, together with the conventional chemical reprogramming process.

**FIGURE 7:**
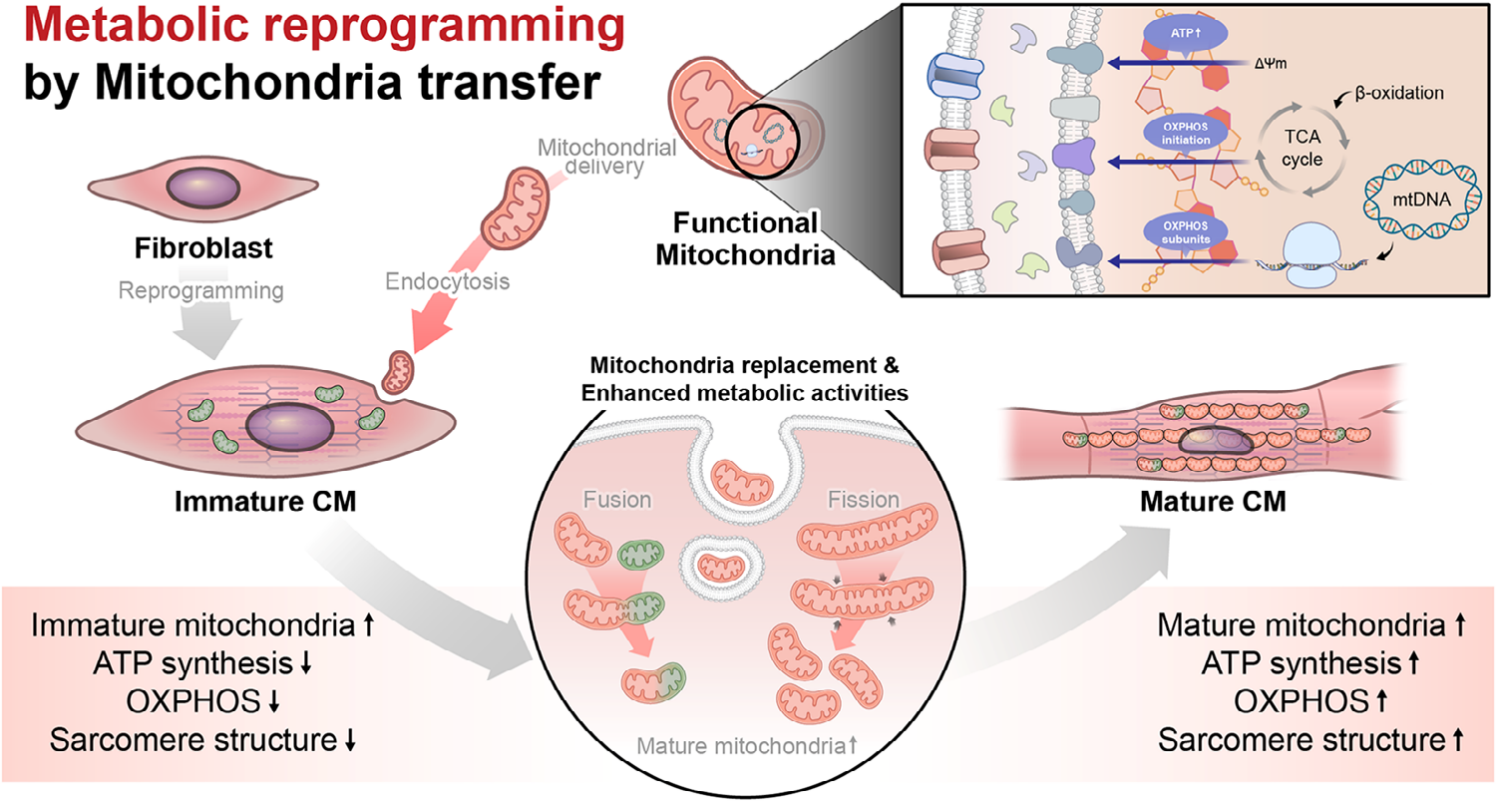
Proposed mechanistic model of mitochondrial delivery inducing metabolic reprograming and maturation of chemically induced cardiomyocyte‐like cells (CiCMs). During the process of direct reprogramming, mitochondrial delivery modulates bioenergetics of immature CMs inducing maturation of the cells. After the treatment of functional mitochondria with robust oxidative phosphorylation (OXPHOS) activity, recipient mitochondria are replaced to mature status, and in turn induces CiCMs metabolically active. As a result, the mitochondria‐treated CiCMs can exhibit enhanced energy production, respiratory activity, and electrophysiological functionality with organized intracellular structures and cardiac‐specific characteristics, resulting in mature CMs.

Our data revealed that mitochondria isolated from heart tissue, compared to those from the brain and liver, had the highest ATP content and the most robust OXPHOS activity, reflecting superior respiratory capacity. This finding is consistent with the known energy demand of the heart, and the essential role of mitochondria in providing the ATP necessary for continuous contraction and relaxation cycles in cardiomyocytes.^69^ This superior mitochondrial performance is presumably due to heart mitochondria having a higher density of OXPHOS components, a characteristic potentially beneficial to metabolic reprogramming of CiCMs. Remarkably, CiCMs treated with mitochondria, particularly those sourced from the heart, displayed more mature phenotypes of cardiomyocyte. This maturity was evident in the increased organization of sarcomeres and more regulated intercellular calcium transients, crucial for excitation‐contraction coupling in mature cardiomyocytes (Figure 7). Furthermore, the mitochondria‐treated CiCMs displayed enhanced metabolic function, confirmed by the increased oxygen consumption rate. Our data aligns with previous research showing that adult cardiomyocytes primarily rely on mitochondrial oxidative metabolism for ATP generation, making the observation of enhanced oxygen consumption in mitochondria‐treated CiCMs an encouraging sign of maturation. Interestingly, we observed mitochondrial fusion events between the introduced mitochondria and those of the recipient cells. This process of mitochondrial fusion and fission is vital for maintaining mitochondrial function and is associated with mature, healthy cells.^21^, ^70^ The increase in the fusion events in our study likely contributes to the enhanced maturation and functionality of CiCMs, indicating a form of mitochondrial “cross‐education” that may facilitate the metabolic transition of these cells. The mechanisms behind metabolic reprogramming, whether it is induced by enhanced functionality of poorly developed native mitochondria, stimulation of mitochondrial biogenesis pathway, or direct increase in mitochondrial number, needs to be unraveled. Furthermore, our findings demonstrate that mitochondrial delivery resulted in structural enhancements and higher expressions of cardiomyocyte‐specific genes in CiCMs. The increase in metabolic gene expression in the mitochondria‐treated groups is particularly interesting, as it suggests that mitochondrial delivery not only improves the existing metabolic profile of CiCMs but might also drive a broader gene expression shift toward a more mature cardiac phenotype. In terms of bioenergetics, our results revealed that the introduction of mitochondria into CiCMs increased oxygen consumption rates, ATP production, maximal respiration, and spare respiratory capacity, without affecting the proton leak. These changes indicate an overall improvement in cellular bioenergetics, a characteristic of mature cardiomyocytes. Additionally, the improved response to the muscarinic agonist carbamylcholine and the more regular and higher amplitude calcium transients in the mitochondria‐treated groups indicate an improved electrical functionality, another sign of enhanced maturation in CiCMs.

While we have proposed mitochondrial transfer‐based metabolic reprogramming as a promising strategy to enhance CiCM maturation, there are several limitations of this study that should be further investigated in future studies. For instance, eventual fate of the exogenous mitochondria internalized within the recipient cells needs to be studied, which will determine the long‐term efficacy of the mitochondrial transfer‐based metabolic reprogramming. Also, a recent finding has suggested that the assimilation of exogenous mitochondria, persistence and role in the recipient cells may differ according to the endogenous mitochondrial function, whether it is respiratory competent or deficient.^71^ However, our study only focused on the behavior of exogenous mitochondria within respiratory competent, differentiating cells. Thus, different observations can be derived from scenarios in which inherent mitochondrial respiratory function is compromised. In addition, we have confirmed metabolic changes and following enhancement in phenotypical maturity of CiCMs after mitochondrial delivery, while future studies on mechanistical investigations are required for further applications in various cell lines and in vivo environments. In addition, the clinical translation of metabolic reprogramming of CiCMs through mitochondrial transplantation has several challenges including mitochondrial storage and safety issues. Due to the narrow time window of mitochondrial activity from the isolation to the treatment, our study ensured mitochondrial transplants be performed directly after isolation. This lack of ability to store isolated mitochondria for a longer period of time significantly limits clinical translation, requiring a methodology that allows storage of viable mitochondria to meet clinical demands. With regard to the safety issues concerning the transfer of mitochondrial DNA (mtDNA), haplotype matching between the donor and the recipient should be considered. In this study, the source for the mitochondrial isolation and the target cells was matched to mouse, but the potential use of autologous, or xenogeneic sources of the mitochondria such as those isolated from mesenchymal stem cells or human cell sources should also be investigated, especially for translational application. Previous studies using animal experiments demonstrated that there was no apparent mismatch effect or detectable immune responses after trans‐species transplantation,^72^, ^73^ but these safety concerns need to be thoroughly addressed before potential clinical applications. Lastly, we have confirmed the stable retention of the H‐Mito‐treated CiCMs in cardiac tissue after 7 days of transplantation, showing potential application in treating cardiac diseases (Figure S8) but this preliminary experiment does not fully test the superiority of H‐Mito CiCMs induced by mitochondrial maturation in a physiologically relevant disease model. Future studies are necessary to thoroughly investigate the regenerative capabilities of H‐Mito CiCMs in vivo, particularly within appropriate disease models.

## 4 CONCLUSIONS

The study offers a potential strategy for enhancing the maturation of CiCMs via metabolic reprogramming. By isolating and delivering mitochondria from adult tissues with high‐energy demand and bioenergetics, we observed that the metabolic profile, structural maturation, and functionality of CiCMs could be substantially improved. This strategy tackles the limitation of CiCMs produced via chemical reprogramming that tend to exhibit immature, fetal‐like cardiomyocyte characteristics which constrain their clinical and research utility. While our results still have several challenges, the strategy of enhancing cellular maturation through metabolic reprogramming opens a novel avenue for enhancing the potential of CiCMs in regenerative medicine, disease modeling, and drug screening.

## 5 MATERIALS AND METHODS

### 5.1 Isolation and culture of primary mouse embryonic fibroblasts (PMEFs)

The Institutional Animal Care and Use Committee of Yonsei University College of Medicine granted approval for the animal experiments under permit number 2022‐0336. All animal experiments were carried out in a facility accredited by the Association for Assessment and Accreditation of Laboratory Animal Care International (AAALAC International). Within this facility, animals had free access to food and water and were maintained on a 12 h light/dark cycle in accordance with established animal protection regulations. PMEFs were isolated from day 13.5 embryos of ICR mice (Orient Bio) following a previously described method.^15^, ^17^ The head, visceral tissues, vertebral column, internal organs, and spinal cord were carefully removed. The remaining tissues were mechanically cut into small pieces. The isolated PMEFs were seeded on a 0.2% (w/v) gelatin (Sigma‐Aldrich)‐coated flask, and cultured in fibroblast culture medium consisting of Dulbecco's modified Eagle's medium (DMEM; #11995065, Themo Fisher Scientific) supplemented with 10% (v/v) fetal bovine serum (FBS; Thermo Fisher Scientific) 1% (v/v) nonessential amino acid (NEAA; Thermo Fisher Scientific) and 1% (v/v) penicillin‐streptomycin (Thermo Fisher Scientific). PMEFs were maintained at 37°C in a 5% CO_2_ incubator, and the medium was replaced every 2 days.

### 5.2 Chemical induction of PMEFs into cardiomyocyte‐like cells using small molecules

For cardiac reprogramming, PMEFs were seeded onto 6‐well plates precoated with Matrigel (Corning Inc., Corning), diluted 1:50 (v/v) in DMEM:Nutrient Mixture F‐12 (DMEM/F12; #11320033, Thermo Fisher Scientific), at a cell density of 2.5 × 10^4^ cells/mL and cultured in fibroblast culture medium. 6 h post‐seeding, the fibroblast culture medium was replaced with cardiac reprogramming medium, composed of DMEM/F12, 15% (v/v) FBS, 5% (v/v) knockout serum replacement (Thermo Fisher Scientific), 1% (v/v) penicillin‐streptomycin, 1% (v/v) NEAA, 1% (v/v) GlutaMax (Thermo Fisher Scientific), 0.1 mM β‐mercaptoethanol (Thermo Fisher Scientific), 15 µM Forskolin (#F‐9929, LC laboratory), 10 µM CHIR99021 (#C‐6556, LC laboratory), 2 µM A83‐01 (#2939, Tocris Bioscience), and 1 µM SC‐1 (#10009557, Cayman Chemical). The chemically induced cardiomyocytes (CiCMs) were cultured at 37°C under 5% CO_2_, with the medium changed every 2 days.

### 5.3 Mitochondrial labeling and imaging

The mitochondria isolated from tissue samples were tagged with 1 nM MitoTracker™ Green FM (#M7514, Thermo Fisher Scientific), which fluoresces at an excitation/emission wavelength of 490/516 nm. The mitochondria were incubated with this label at 4°C for 10 min. Subsequently, the mitochondria were rinsed thrice using cold PBS (Sigma‐Aldrich). Native mitochondria were labeled with 1 nM MitoTracker™ Red CMXROS (#7512, Thermo Fisher Scientific), with fluorescence properties of 579/599 nm excitation/emission, for 10 min at 37°C. Post‐incubation for 24 h of the isolated mitochondria with cells undergoing direct cardiac reprogramming chemically treated for 9 days, these were cleansed thrice using PBS. The nuclei were marked with 4′,6‐diamidino‐2‐phenylindole (DAPI; TCI Chemicals). A confocal microscope was employed to capture the process of mitochondrial internalization (Zeiss LSM 980, Carl Zeiss).

### 5.4 Immunofluorescent staining

Cells and heart tissues were fixed in a 10% formalin solution (Sigma‐Aldrich) for 10 min at room temperature or overnight at 4°C, respectively. For cryo‐sectioning of heart tissues, the samples were incubated in 30% (v/v) sucrose (Sigma‐Aldrich) solution in PBS overnight at 4°C. The samples were then embedded in optical cutting temperature (OCR) compound (CellPath), frozen at –80°C, and sliced into 7 µm sections for immunochemical staining. The cells and sectioned heart tissues were then permeabilized by 0.1% Triton X‐100 (Sigma‐Aldrich) for 10 min, and the sectioned heart tissues were treated with 0.3% Triton X‐100 for another 20 min. Samples were blocked with 5% BSA (Fraction V, MP Biomedicals) for 1 h, before overnight incubation at 4°C with primary antibodies. After several washes with PBS, secondary antibodies conjugated with Alexa‐Fluor 594 goat antimouse IgG (1:200, #A11005, Thermo Fisher Scientific) were applied. Nuclei were subsequently stained using DAPI. The antibodies used in this study include mouse monoclonal anti‐α‐actinin (1:500, #A7811, Sigma‐Aldrich), mouse monoclonal anticardiac Troponin T (cTnT; 1:200, #MA5‐12960, Thermo Fisher Scientific), and FITC‐conjugated wheat germ agglutinin (WGA; 5 µg/mL, #L4895, Sigma‐Aldrich). The whole‐mount staining procedure was carried out on all samples, which were subsequently imaged using confocal microscopy (LSM 980, Carl Zeiss).

### 5.5 Quantitative real‐time polymerase chain reaction (qRT‐PCR)

The mRNA expression levels of cardiac markers (Mef2c, Mesp1, Nkxx2‐5, Scn5a, and Myh7) and metabolic markers (PPARα, Cpt1b, Tfam, and Nfe2l2) in CiCM were quantified through qRT‐PCR. RNA was isolated from each sample using an RNA extraction kit (Takara Bio), from which cDNA was synthesized using a cDNA synthesis kit (Takara Bio). The qRT‐PCR procedure was performed with TaqMan Fast Universal PCR Master Mix (Applied Biosystems) on a QuantStudio3TM Real‐Time PCR system (Applied Biosystems). The primers used included mouse *Mef2c* (Mm01340842_m1), mouse *Mesp1* (Mm00801883‐g1), mouse *Nkx2‐5* (Mm01309813_s1), mouse *Scn5a* (Mm01342518_m1), mouse *Myh7* (Mm00600555_m1), *PPARα* (Mm00440939_m1), mouse *Cpt1b* (Mm00487191_g1), mouse *Tfam* (Mm00447485_m1), and mouse *Nfe2l2* (Mm00477784_m1). The relative gene expression levels were calculated using the cycle threshold method, with normalization to mouse *Gapdh* (Mm99999915_g1) serving as the endogenous control gene.

### 5.6 Statistical analysis

All quantitative data are presented as means ± standard deviation (SD). The “*n*” values specified in the figure legends denote biological replicates. Statistical differences between groups were analyzed using a two‐tailed unpaired *t*‐test or two‐way analysis of variance (ANOVA) followed by Bonferroni or Tukey with Prism 8 software (GraphPad). A *p*‐value ˂0.05 was considered statistically significant.

## AUTHOR CONTRIBUTIONS

*Conception and design*: Mikyung Shin, Yoonhee Jin, and Jung Seung Lee. *Development of methodology*: Yena Nam, Yoonji Song, Seung Ju Seo, Ga Ryang Ko, Seung Hyun Lee, Eunju Cha, Su Min Kwak, and Sumin Kim. *Acquisition of data*: Yena Nam and Yoonji Song. *Writing and review, and/or revision of the manuscript*: Yena Nam, Yoonji Song, Yoonhee Jin, and Jung Seung Lee. *Administrative, technical, or material support*: Mikyung Shin, Yoonhee Jin and Jung Seung Lee. All authors have read and approved the final manuscript.

## CONFLICT OF INTEREST STATEMENT

The authors declare no conflicts of interest.

## ETHICS STATEMENT

The Institutional Animal Care and Use Committee of Yonsei University College of Medicine and Institutional Animal Care and Use Committee of Sungkyunkwan University granted approval for the animal experiments under permit number (Yonsei University: 2022‐0336) and (Sungkyunkwan University: 2023‐11‐38‐1), respectively. All animal experiments were carried out in a facility accredited by the Association for Assessment and Accreditation of Laboratory Animal Care International (AAALAC International).

## Supporting information



Supporting Information

## Data Availability

The data that supports the conclusions can be obtained from the corresponding authors upon reasonable request via email.

## References

[1] Ieda M , Fu JD , Delgado‐Olguin P , et al. Direct reprogramming of fibroblasts into functional cardiomyocytes by defined factors. Cell. 2010;142(3):375‐386. 20691899 10.1016/j.cell.2010.07.002 PMC2919844

[2] Fu YB , Huang CW , Xu XX , et al. Direct reprogramming of mouse fibroblasts into cardiomyocytes with chemical cocktails. Cell Res. 2015;25(9):1013‐1024. 26292833 10.1038/cr.2015.99 PMC4559819

[3] Wang H , Yang Y , Liu J , Qian L . Direct cell reprogramming: approaches, mechanisms and progress. Nat Rev Mol Cell Biol. 2021;22(6):410‐424. 33619373 10.1038/s41580-021-00335-z PMC8161510

[4] Yamakawa H , Ieda M , Cardiac regeneration by direct reprogramming in this decade and beyond. Inflamm Regen. 2021;41(1):20. 34193320 10.1186/s41232-021-00168-5 PMC8247073

[5] Tang Y , Aryal S , Geng X , et al. TBX20 Improves Contractility and mitochondrial function during direct human cardiac reprogramming. Circulation. 2022;146(20):1518‐1536. 36102189 10.1161/CIRCULATIONAHA.122.059713 PMC9662826

[6] Mohamed TMA , Stone NR , Berry EC , et al. Chemical enhancement of in vitro and in vivo direct cardiac reprogramming. Circulation. 2017;135(10):978‐995. 27834668 10.1161/CIRCULATIONAHA.116.024692 PMC5340593

[7] Wolfram JA , Donahue JK . Gene therapy to treat cardiovascular disease. J Am Heart Assoc. 2013;2(4):e000119. 23963752 10.1161/JAHA.113.000119 PMC3828796

[8] Cao N , Huang Y , Zheng JS , et al. Conversion of human fibroblasts into functional cardiomyocytes by small molecules. Science. 2016;352(6290):1216‐1220. 27127239 10.1126/science.aaf1502

[9] Singh VP , Pinnamaneni JP , Pugazenthi A , et al. Enhanced generation of induced cardiomyocytes using a small‐molecule cocktail to overcome barriers to cardiac cellular reprogramming. J Am Heart Assoc. 2020;9(12):e015686. 32500803 10.1161/JAHA.119.015686 PMC7429035

[10] He XY , Liang JL , Paul C , Huang W , Dutta S , Wang YG . Advances in cellular reprogramming‐based approaches for heart regenerative repair. Cells. 2022;11(23):3914. 36497171 10.3390/cells11233914 PMC9740402

[11] Tani H , Sadahiro T , Ieda M . Direct cardiac reprogramming: a novel approach for heart regeneration. Int J Mol Sci. 2018;19(9):2629. 30189626 10.3390/ijms19092629 PMC6165160

[12] Takeda Y , Harada Y , Yoshikawa T , Dai P . Chemical compound‐based direct reprogramming for future clinical applications. Bioscience Rep. 2018;38:BSR20171650. 10.1042/BSR20171650 PMC5938430 29739872

[13] Sia J , Yu P , Srivastava D , Li S . Effect of biophysical cues on reprogramming to cardiomyocytes. Biomaterials. 2016;103:1‐11. 27376554 10.1016/j.biomaterials.2016.06.034

[14] Li Y , Dal‐Pra S , Mirotsou M , et al. Tissue‐engineered 3‐dimensional (3D) microenvironment enhances the direct reprogramming of fibroblasts into cardiomyocytes by microRNAs. Sci Rep. 2016;6(1):38815. 27941896 10.1038/srep38815 PMC5150639

[15] Jin Y , Lee JS , Kim J , et al. Three‐dimensional brain‐like microenvironments facilitate the direct reprogramming of fibroblasts into therapeutic neurons. Nat Biomed Eng. 2018;2(7):522‐539. 30948831 10.1038/s41551-018-0260-8

[16] Min S , Lee H‐J , Jin Y , et al. Biphasic electrical pulse by a micropillar electrode array enhances maturation and drug response of reprogrammed cardiac spheroids. Nano Lett. 2020;20(10):6947‐6956. 32877191 10.1021/acs.nanolett.0c01141

[17] Jin Y , Kim H , Min S , et al. Three‐dimensional heart extracellular matrix enhances chemically induced direct cardiac reprogramming. Sci Adv. 8(50):eabn5768. 36516259 10.1126/sciadv.abn5768 PMC9750148

[18] San‐Millán I . The key role of mitochondrial function in health and disease. Antioxidants. 2023;12(4):782. 37107158 10.3390/antiox12040782 PMC10135185

[19] Vakifahmetoglu‐Norberg H , Ouchida AT , Norberg E . The role of mitochondria in metabolism and cell death. BBRC. 2017;482(3):426‐431. 28212726 10.1016/j.bbrc.2016.11.088

[20] Tzameli I . The evolving role of mitochondria in metabolism. Trends Endocrinol Metab. 2012;23(9):417‐419. 22901785 10.1016/j.tem.2012.07.008

[21] Garbern JC , Lee RT . Mitochondria and metabolic transitions in cardiomyocytes: lessons from development for stem cell‐derived cardiomyocytes. Stem Cell Res Ther. 2021;12(1):177. 33712058 10.1186/s13287-021-02252-6 PMC7953594

[22] Ding QQ , Qi YX , Tsang SY . Mitochondrial biogenesis, mitochondrial dynamics, and mitophagy in the maturation of cardiomyocytes. Cells. 2021;10(9):2463. 34572112 10.3390/cells10092463 PMC8466139

[23] Lehman JJ , Barger PM , Kovacs A , Saffitz JE , Medeiros DM , Kelly DP . Peroxisome proliferator–activated receptor γ coactivator‐1 promotes cardiac mitochondrial biogenesis. JCI. 2000;106(7):847‐856. 11018072 10.1172/JCI10268 PMC517815

[24] Morita Y , Tohyama S . Metabolic regulation of cardiac differentiation and maturation in pluripotent stem cells: a lesson from heart development. JMA J. 2020;3(3):193‐200. 33150253 10.31662/jmaj.2020-0036 PMC7590396

[25] Correia M , Santos F , Ferreira RD , Ferreira R , de Jesus BB , Nóbrega‐Pereira S . Metabolic determinants in cardiomyocyte function and heart regenerative strategies. Metabolites. 2022;12(6):500. 35736435 10.3390/metabo12060500 PMC9227827

[26] Liu D , Gao Y , Liu J , et al. Intercellular mitochondrial transfer as a means of tissue revitalization. Signal Transduct Target Ther. 2021;6(1):65. 33589598 10.1038/s41392-020-00440-z PMC7884415

[27] Caicedo A , Aponte PM , Cabrera F , Hidalgo C , Khoury M . Artificial Mitochondria transfer: current challenges, advances, and future applications. Stem Cells Int. 2017;2017(1):7610414. 28751917 10.1155/2017/7610414 PMC5511681

[28] Acquistapace A , Bru T , Lesault PF , et al. Human Mesenchymal stem cells reprogram adult cardiomyocytes toward a progenitor‐like state through partial cell fusion and mitochondria transfer. Stem Cells. 2011;29(5):812‐824. 21433223 10.1002/stem.632 PMC3346716

[29] Ikeda G , Santoso MR , Tada Y , et al. Mitochondria‐rich extracellular vesicles from autologous stem cell‐derived cardiomyocytes restore energetics of ischemic myocardium. J Am Coll Cardiol. 2021;77(8):1073‐1088. 33632482 10.1016/j.jacc.2020.12.060 PMC8626617

[30] Sadeesh EM , Singla N , Lahamge MS , Kumari S , Ampadi AN , Anuj M . Tissue heterogeneity of mitochondrial activity, biogenesis and mitochondrial protein gene expression in buffalo. Mol Biol Rep. 2023;50(6):5255‐5266. 37140692 10.1007/s11033-023-08416-2

[31] Nascimento‐Dos‐Santos G , de‐Souza‐Ferreira E , Lani R , et al. Neuroprotection from optic nerve injury and modulation of oxidative metabolism by transplantation of active mitochondria to the retina. BBA Mol Basis Dis. 2020;1866(5):165686. 10.1016/j.bbadis.2020.165686 31953215

[32] Bak S , Leon IR , Jensen ON , Hojlund K . Tissue specific phosphorylation of mitochondrial proteins isolated from rat liver, heart muscle, and skeletal muscle. J Proteome Res. 2013;12(10):4327‐4339. 23991683 10.1021/pr400281r

[33] Chen W , Shi K , Chu B , Wei X , Qian Z . Mitochondrial surface engineering for multidrug resistance reversal. Nano Lett. 2019;19(5):2905‐2913. 30935203 10.1021/acs.nanolett.8b05188

[34] Zhou W , Zhao Z , Yu Z , Hou Y , Keerthiga R , Fu A . Mitochondrial transplantation therapy inhibits the proliferation of malignant hepatocellular carcinoma and its mechanism. Mitochondrion. 2022;65:11‐22. 35504558 10.1016/j.mito.2022.04.004

[35] Chang J‐C , Chang H‐S , Wu Y‐C , et al. Antitumor actions of intratumoral delivery of membrane‐fused mitochondria in a mouse model of triple‐negative breast cancers. Onco Targets Ther. 2020;13:5241‐5255. 32606744 10.2147/OTT.S238143 PMC7294573

[36] Sun X , Chen H , Gao R , et al. Intravenous Transplantation of an ischemic‐specific peptide‐tpp‐mitochondrial compound alleviates myocardial ischemic reperfusion injury. ACS Nano. 2023;17(2):896‐909. 36625783 10.1021/acsnano.2c05286 PMC9878726

[37] Monteiro LdB , Davanzo GG , de Aguiar CF , Moraes‐Vieira PMM . Using flow cytometry for mitochondrial assays. MethodsX. 2020;7:100938. 32551241 10.1016/j.mex.2020.100938 PMC7289760

[38] Haseda K , Kanematsu K , Noguchi K , Saito H , Umeda N , Ohta Y . Significant correlation between refractive index and activity of mitochondria: single mitochondrion study. Biomed Opt Express. 2015;6(3):859‐869. 25798310 10.1364/BOE.6.000859 PMC4361440

[39] Adams RA , Liu Z , Hsieh C , et al. Structural Analysis of mitochondria in cardiomyocytes: insights into bioenergetics and membrane remodeling. CIMB. 2023;45(7):6097‐6115. 37504301 10.3390/cimb45070385 PMC10378267

[40] Smith RA , Porteous CM , Gane AM , Murphy MP . Delivery of bioactive molecules to mitochondria in vivo. Proc Natl Acad Sci USA. 2003;100(9):5407‐5412. 12697897 10.1073/pnas.0931245100 PMC154358

[41] Michelakis ED . Mitochondrial medicine. Circulation. 2008;117(19):2431‐2434. 18474822 10.1161/CIRCULATIONAHA.108.775163

[42] Sha L , Daitoku H , Araoi S , et al. Asymmetric arginine dimethylation modulates mitochondrial energy metabolism and homeostasis in caenorhabditis elegans. Mol Cell Biol. 2017;37(6):e00504. 27994012 10.1128/MCB.00504-16 PMC5335503

[43] Fernandez‐Vizarra E , Enriquez JA , Perez‐Martos A , Montoya J , Fernandez‐Silva P . Tissue‐specific differences in mitochondrial activity and biogenesis. Mitochondrion. 2011;11(1):207‐213. 20933104 10.1016/j.mito.2010.09.011

[44] Meeusen S , McCaffery JM , Nunnari J . Mitochondrial fusion intermediates revealed in vitro. Science. 2004;305(5691):1747‐1752. 15297626 10.1126/science.1100612

[45] Ni H‐M , Williams JA , Ding W‐X . Mitochondrial dynamics and mitochondrial quality control. Redox Biol. 2015;4:6‐13. 25479550 10.1016/j.redox.2014.11.006 PMC4309858

[46] Ryu S‐W , Han EC , Yoon J , Choi C . The Mitochondrial fusion‐related proteins Mfn2 and OPA1 are transcriptionally induced during differentiation of bone marrow progenitors to immature dendritic cells. Mol Cells. 2015;38(1):89‐94. 25387754 10.14348/molcells.2015.2285 PMC4314123

[47] Dallman PR , Goodman JR . Enlargement of mitochondrial compartment in iron and copper deficiency. Blood. 1970;35(4):496‐505. 4315322

[48] Bustos RI , Jensen EL , Ruiz LM , et al. Copper deficiency alters cell bioenergetics and induces mitochondrial fusion through up‐regulation of MFN2 and OPA1 in erythropoietic cells. BBRC. 2013;437(3):426‐432. 23831624 10.1016/j.bbrc.2013.06.095

[49] Schraps N , Tirre M , Pyschny S , et al. Cardiomyocyte maturation alters molecular stress response capacities and determines cell survival upon mitochondrial dysfunction. Free Radic Biol Med. 2024;213:248‐265. 38266827 10.1016/j.freeradbiomed.2024.01.034

[50] Georgiadi A , Boekschoten MV , Muller M , Kersten S . Detailed transcriptomics analysis of the effect of dietary fatty acids on gene expression in the heart. Physiol Genomics. 2012;44(6):352‐361. 22274564 10.1152/physiolgenomics.00115.2011

[51] Kersten S . Integrated physiology and systems biology of PPARα. Mol Metab. 2014;3(4):354‐371. 24944896 10.1016/j.molmet.2014.02.002 PMC4060217

[52] Durgan DJ , Smith JK , Hotze MA , et al. Distinct transcriptional regulation of long‐chain acyl‐CoA synthetase isoforms and cytosolic thioesterase 1 in the rodent heart by fatty acids and insulin. Am J Physiol Heart Circ Physiol. 2006;290(6):H2480‐H2497. 16428347 10.1152/ajpheart.01344.2005

[53] van der Lee KAJM , Vork MM , De Vries JE , et al. Long‐chain fatty acid‐induced changes in gene expression in neonatal cardiac myocytes. JLR. 2000;41(1):41‐47. 10627500

[54] He L , Kim T , Long QQ , et al. Carnitine Palmitoyltransferase‐1b deficiency aggravates pressure overload‐induced cardiac hypertrophy caused by lipotoxicity. Circulation. 2012;126(14):1705‐1716. 22932257 10.1161/CIRCULATIONAHA.111.075978 PMC3484985

[55] Eaton S , Bartlett K , Quant PA . Carnitine Palmitoyl transferase i and the control of β‐oxidation in heart mitochondria. BBRC. 2001;285(2):537‐539. 11444876 10.1006/bbrc.2001.5201

[56] Li X , Wu F , Gunther S , et al. Inhibition of fatty acid oxidation enables heart regeneration in adult mice. Nature. 2023;622(7983):619‐626. 37758950 10.1038/s41586-023-06585-5 PMC10584682

[57] Ikeuchi M , Matsusaka H , Kang D , et al. Overexpression of mitochondrial transcription factor A ameliorates mitochondrial deficiencies and cardiac failure after myocardial infarction. Circulation. 2005;112(5):683‐690. 16043643 10.1161/CIRCULATIONAHA.104.524835

[58] Ikeda M , Ide T , Fujino T , et al. Overexpression of TFAM or Twinkle increases mtDNA copy number and facilitates cardioprotection associated with Limited mitochondrial oxidative stress. PLOS ONE. 2015;10(3):e0119687. 25822152 10.1371/journal.pone.0119687 PMC4379048

[59] Suarez J , Hu Y , Makino A , Fricovsky E , Wang H , Dillmann WH . Alterations in mitochondrial function and cytosolic calcium induced by hyperglycemia are restored by mitochondrial transcription factor A in cardiomyocytes. Am J Physiol Cell Ph. 2008;295(6):C1561‐C1568. 10.1152/ajpcell.00076.2008 PMC2603561 19060297

[60] Kang I , Chu CT , Kaufman BA . The mitochondrial transcription factor TFAM in neurodegeneration: emerging evidence and mechanisms. Febs Lett. 2018;592(5):793‐811. 29364506 10.1002/1873-3468.12989 PMC5851836

[61] Dinkova‐Kostova AT , Abramov AY . The emerging role of Nrf2 in mitochondrial function. Free Radic Biol Med. 2015;88:179‐188. 25975984 10.1016/j.freeradbiomed.2015.04.036 PMC4726722

[62] Limas CJ , Limas C . Carbachol induces desensitization of cardiac β‐adrenergic receptors through muscarinic M1 receptors. BBRC. 1985;128(2):699‐704. 2986622 10.1016/0006-291x(85)90103-2

[63] Banach K , Halbach MD , Hu P , Hescheler J , Egert U . Development of electrical activity in cardiac myocyte aggregates derived from mouse embryonic stem cells. Am J Physiol Heart Circ Physiol. 2003;284(6):H2114‐H2123. 12573993 10.1152/ajpheart.01106.2001

[64] He JQ , Ma Y , Lee Y , Thomson JA , Kamp TJ . Human embryonic stem cells develop into multiple types of cardiac myocytes—action potential characterization. Circ Res. 2003;93(1):32‐39. 12791707 10.1161/01.RES.0000080317.92718.99

[65] Oerlemans MI , Koudstaal S , Chamuleau SA , de Kleijn DP , Doevendans PA , Sluijter JP . Targeting cell death in the reperfused heart: pharmacological approaches for cardioprotection. Int J Cardiol. 2013;165(3):410‐422. 22459400 10.1016/j.ijcard.2012.03.055

[66] Peters MC , Maas RGC , van Adrichem I , et al. Metabolic maturation increases susceptibility to hypoxia‐induced damage in human iPSC‐derived cardiomyocytes. Stem Cells Transl Med. 2022;11(10):1040‐1051. 36018047 10.1093/stcltm/szac061 PMC9585948

[67] Huang T , Lin R , Su Y , et al. Efficient intervention for pulmonary fibrosis via mitochondrial transfer promoted by mitochondrial biogenesis. Nat Commun. 2023;14(1):5781. 37723135 10.1038/s41467-023-41529-7 PMC10507082

[68] Lee JM , Hwang JW , Kim MJ , et al. Mitochondrial Transplantation Modulates Inflammation And Apoptosis, Alleviating Tendinopathy Both In Vivo And In Vitro. Antioxidants (Basel). 2021;10(5):696. 33925007 10.3390/antiox10050696 PMC8146308

[69] Torrealba N , Aranguiz P , Alonso C , Rothermel BA , Lavandero S . Mitochondria in structural and functional cardiac remodeling. Adv Exp Med Biol. 2017;982:277‐306. 28551793 10.1007/978-3-319-55330-6_15

[70] Tan Y , Xia F , Li L , et al. Novel insights into the molecular features and regulatory mechanisms of mitochondrial dynamic disorder in the pathogenesis of cardiovascular disease. Oxid Med Cell Longev. 2021;2021:6669075. 33688392 10.1155/2021/6669075 PMC7914101

[71] Lin R‐Z , Im G‐B , Luo AC , et al. Mitochondrial transfer mediates endothelial cell engraftment through mitophagy. Nature. 2024;629(8012):660‐668. 38693258 10.1038/s41586-024-07340-0 PMC11574736

[72] Cannon MV , Takeda K , Pinkert CA . Mitochondrial biology in reproduction. Reprod Med Biol. 2011;10(4):251‐258. 29662358 10.1007/s12522-011-0101-x PMC5892994

[73] Chang J‐C , Wu S‐L , Liu K‐H , et al. Allogeneic/xenogeneic transplantation of peptide‐labeled mitochondria in Parkinson's disease: restoration of mitochondria functions and attenuation of 6‐hydroxydopamine–induced neurotoxicity. Transl Res. 2016;170:40‐56. e3. 26730494 10.1016/j.trsl.2015.12.003

