# Supplementary material for: Metabolic reprogramming via mitochondrial delivery for enhanced maturation of chemically induced cardiomyocyte‐like cells
Source: MedComm (2020). 2024 Nov 28;5(12):e70005. doi: 10.1002/mco2.70005 (PMC11604293; doi:10.1002/mco2.70005)
Supplement: Supplementary file 1 — Supporting Information [file MCO2-5-e70005-s001.docx]

Supporting Information

Metabolic Reprogramming via Mitochondrial Delivery for Enhanced Maturation of Chemically Induced Cardiomyocyte-like Cells

Yena Nam, Yoonji Song, Seung Ju Seo, Ga Ryang Ko, Seung Hyun Lee, Eunju Cha, Su Min Kwak, Sumin Kim, Mikyung Shin, Yoonhee Jin^*^, Jung Seung Lee^*^

Y. Nam, S. J. Seo, E. Cha, Y. Jin

Department of Physiology, Graduate School of Medical Science, Brain Korea 21 Project, Yonsei University College of Medicine, Seoul 03722, Republic of Korea

E-mail: [yoonheejin@yuhs.ac](mailto:yoonheejin@yuhs.ac)

S. M. Kwak, Y. Jin

Department of Medicine, College of Medicine, Yonsei University Graduate School, Seoul 03722, Republic of Korea

Y. Song, M. Shin, J. S. Lee

Department of Biomedical Engineering, Sungkyunkwan University (SKKU), Suwon 16419, Republic of Korea

E-mail: [jungseunglee@skku.edu](mailto:jungseunglee@skku.edu)

G. R. Ko, S. H. Lee, S. Kim, M. Shin, J. S. Lee

Department of Intelligent Precision Healthcare Convergence, Sungkyunkwan University (SKKU), Suwon 16419, Republic of Korea

M. Shin

Center for Neuroscience Imaging Research, Institute for Basic Science (IBS), Suwon 16419, Republic of Korea

J. S. Lee

Department of MetaBioHealth, Sungkyunkwan University (SKKU), Suwon 16419, Republic of Korea

Materials and Methods

*Isolation of mitochondria*: Mitochondria were isolated from ICR mouse (5-week-old females, Orientbio) tissues using differential centrifugation as described previously,^73^ with some modifications. After removal, all tissues were immediately washed with PBS and placed in ice-cold Homogenizing Buffer (Sucrose 300 mM, HEPES 10 mM, ethylene glycol bis(2-aminoethyl ether)-N,N,N’,N’-tetraacetic acid [EGTA] 1 mM, pH = 7.2), supplemented with bovine serum albumin (BSA; 20 mg/ml). Then, all tissues were homogenized and filtered through 100 𝜇m and 40 𝜇m cell strainer. Homogenates were centrifuged at 700 g for 10 min at 4℃. Then, the supernatant was centrifuged at 3,220 g for 20 min at 4℃. The pellets were washed in PBS, and again filtered through 5 𝜇m filter, centrifuged at 3,000 g for 5 min at 4℃. The pellets were resuspended in PBS and centrifuged at 3,000 g for 5 min at 4℃ twice. Final mitochondrial pellets were collected and resuspended in different buffer solutions according to subsequent analysis.

*Characterization of isolated mitochondria*: The size of isolated mitochondria was measured by a Multisizer 4e Coulter Counter (Beckman Coulter, Brea, CA, USA) and the zeta potential was determined by dynamic light scattering (DLS) analysis (Zetasizer Lab, Malvern Panalytical, Malvern, UK). The protein content in mitochondrial preparations was determined using Bicinchoninic acid (BCA) Protein Assay Kit (Takara Bio, Kusatsu, Japan). The assay was performed according to the manufacturer’s protocol and the optical density was measured at a wavelength of 562 nm with a microplate reader (Synergy HTX, BioTek, Winooski, VT, USA).

*Flow cytometry*: The purity of isolated mitochondria and mitochondrial delivery efficiency into CiCMs were measured using flow cytometry analysis. To analyze mitochondrial isolation purity, the isolated mitochondria were stained with 200 nM MitoTracker™ Red CMXROS (#7512, Thermo Fisher Scientific) at 37℃ for 30 min in the dark. After incubation, the mitochondria were washed with PBS at 3,000 g for 5 min at 4℃ three times and diluted in ice-cold, filtered PBS. To analyze mitochondrial delivery efficiency, the mitochondria isolated from brain, liver, and heart tissues were stained with 1 nM MitoTracker™ Green FM (#M7514, Thermo Fisher Scientific) before being treated with CiCMs. To assess the efficiency of mitochondrial transfer into the cells, the cells were collected through trypsinization and washed with ice-cold PBS. Subsequently, the collected cells were fixed with 4% paraformaldehyde for 10 min. The samples were then washed and diluted in PBS, and analyzed using the CytoFLEX (Beckman Coulter).

*Transmission electron microscopy (TEM) of isolated mitochondria and CiCMs*: To examine the morphology of isolated mitochondria and structural characteristics of CiCMs, both with and without mitochondrial treatment, samples were prepared through a series of processing steps, which involved purification, fixation, post-fixation, dehydration, infiltration, and embedding. The samples were then sectioned into semi-thin slices, and these sections underwent staining and imaging based on a previously established method.^17^ Images were captured with a transmission electron microscope (HT7800, HITACHI, Tokyo, Japan) at an acceleration voltage of 80 kV equipped with a RC camera.

*ATP assay*: The ATPlite Luminescence Assay Kit (PerkinElmer, Waltham, MA, USA) was used for the measurement of mitochondrial ATP levels, according to the manufacturer’s instructions. The luminescent signals were measured with the SpectraMax iD3 Microplate Reader (Molecular Devices, San Jose, CA, USA).

*Extracellular oxygen consumption rate assay*: The extracellular oxygen consumption rate (OCR) of isolated mitochondria was detected with a commercially available assay kit (#ab197243, Abcam, Cambridge, UK). The isolated mitochondria were diluted in Measurement Buffer (Sucrose 250 mM, KCl 15 mM, EGTA 1 mM, MgCl_2_ 5 mM, K_2_HPO_4_ 30 mM, pH = 7.4) and basal state (State 2) substrate solution was added to sample wells. High-sensitivity mineral oil preheated at 37℃ was added to each well and the fluorescence signals were measured using a microplate reader (Molecular Devices, USA) for 1 h at 30℃. The signals were collected at 1.5 min intervals at excitation/emission (Ex/Em) = 380/650 nm.

*Cytochrome c oxidase activity assay*: The enzymatic activity of cytochrome *c* oxidase was measured spectrophotometrically by the decrease in an absorbance at 550 nm as the oxidation of reduced cytochrome *c*. Mitochondrial samples were diluted in 1X Assay Buffer (KH_2_PO_4_ 10 mM, Sucrose 250 mM, pH = 7.0) supplemented with 1X Enzyme Buffer (n-Dodecyl β-D-maltoside 1 mM in 1X Assay Buffer) and 0.1 mM reduced cytochrome *c* solution were added to each sample. Immediately after mixing, the optical density was measured at 5 s intervals.

*Immunoblotting*: Mitochondrial proteins were prepared in radioimmunoprecipitation assay buffer (RIPA) lysis buffer (1% Triton X-100, 0.1% sodium dodecyl sulfate (SDS), 1% sodium deoxycholate, NaCl 150 mM, Tris 50 mM, ethylene-diamine-tetraacetic acid [EDTA] 2 mM, pH = 7.4) containing 1X Xpert Duo Inhibitor Cocktail Solution (GenDEPOT, Baker, TX, USA). To detect the expression of mitochondrial dynamics and OXPHOS complex subunit protein in a cellular level, CiCMs samples were washed twice with ice-cold PBS and then lysed in RIPA lysis buffer (Sigma-Aldrich) containing a protease inhibitor cocktail (Roche, Basel, Switzerland). After 30 min incubation at 4℃, the samples were centrifuged at 16,000 g for 20 min at 4℃ and the supernatant was collected into a fresh tube. Protein concentrations were determined using the BCA Protein Assay Kit (Takara Bio). Extracted proteins were resolved on sodium dodecyl sulfate-polyacrylamide gel electrophoresis (SDS-PAGE) gel and blotted onto polyvinylidene fluoride (PDVF) membranes. Transferred protein was blocked with 2% skim milk solution in TBST buffer (Tris-buffered saline, 0.1% Tween-20) and the membrane was incubated with primary antibody (1:1000) overnight at 4℃. Then, appropriate horseradish peroxidase-conjugated secondary antibody (1:5000) was reacted with the membrane at room temperature for 1 h. The primary antibodies were used against mitochondrial OXPHOS complex I to V (#ab110413, Abcam), mitofusin 2 (MFN2; #9482, Cell Signaling Technology, Danvers, MA, USA), optic atrophy 1 (OPA1; NBP2-59770, Novus Biologicals, Littleton, CO, USA), dynamin-related protein 1 (DRP1; #8570, Cell Signaling Technology) and anti-TOM20 (#42406, Cell Signaling Technology) and β-actin (#3700, Cell Signaling Technology) were used as loading controls. For secondary antibody, goat anti-rabbit IgG (#65-6120, Thermo Fisher Scientific) or goat anti-mouse IgG (#31430, Thermo Fisher Scientific) was used. The membrane was soaked in WSE-7120 EzWestLumi plus reagent A and B (ATTO, Tokyo, Japan) in a 1:1 ration and detected using a WSE-6200 LuminoGraph II (ATTO). The individual protein band density was quantified with ImageJ software (NIH, USA).

*Mitochondrial transfer*: Isolated mitochondria from liver, brain, and heart tissues were transferred to CiCMs one day after passaging, with the concentration of added mitochondria at 100 μg/ml. The medium was replaced 24 h after mitochondrial transfer, and subsequently refreshed every two days.

*Mitochondrial DNA (mtDNA) copy number quantification assay*: The mitochondrial delivery efficiency into CiCMs was quantified using quantitative real-time polymerase chain reaction (qRT-PCR). DNA was isolated from each sample using the SpeeDNA Isolation Kit (#MB6918, ScienCell Research Laboratories, Carlsbad, CA, USA) and mtDNA from each sample was detected with the Absolute Mouse Mitochondrial DNA Copy Number Quantification qPCR Assay Kit (#M8948, ScienCell Research Laboratories), according to the manufacturer’s instructions. qPCR was performed on a QuantStudio3TM Real-Time PCR system (Applied Biosystems, Waltham, MA, USA).

*Oxygen consumption rate (OCR) and proton efflux rate (PER) assessment*: To explore the bioenergetic implications of mitochondrial delivery, the OCR and PER were assessed using a Seahorse XFe 96 Analyzer (Agilent Technologies Inc., Santa Clara, CA, USA). Initially, CiCMs that had received mitochondrial treatment for 10 days were seeded on Matrigel-precoated Seahorse XFe 96 microplates (diluted 1:50, v/v) at a density of 5×10^4^ cells per well. To prepare for the experiment, sensor cartridges were hydrated with XF Calibrant Solution (pH 7.4) and incubated them at 37℃ in a non-CO_2_ incubator for 12 h, one day before the experiment. After a 12 h cell-seeding period, the culture medium was replaced with XF DMEM Based Media (pH 7.4), containing 17.5 mM glucose, 0.5 mM pyruvate, and 2.5 mM glutamine, after washing the cells twice with assay media. For OCR assessment, the assay cartridge was pre-loaded with 2.5 µM oligomycin, 1 µM carbonyl cyanide-p-trifluoromethoxyphenylhydrazone (FCCP), and a mixture of 2.5 µM rotenone and 2.5 µM antimycin A. For PER assessment, a mixture of rotenone and antimycin A, each at concentration of 2.5 µM, along with 25 mM 2-Deoxy-D-glucose (2-DG) at final concentration, were used.

*Calcium imaging*: To assess intracellular calcium transients in each CiCM group, calcium imaging based on fluorescent indicators was conducted. Cells were stained with 1 μM Fluo-4 AM (Thermo Fisher Scientific) for 30 min at 37℃. After washing with basal medium, consecutive images of calcium transients were recorded using a confocal microscope (LSM 710, Carl Zeiss). Regions of interest were selected and changes in fluorescence intensity were analyzed with ZEN software (Carl Zeiss). We manually calculated the normalized peak amplitude and frequency. For drug testing, CiCMs were exposed to Carbamoylcholine chloride (Sigma-Aldrich) at concentrations of 10 nM and 100 nM. We monitored alterations in calcium transients in the CiCMs post drug administration.

*Hypoxia Induction*: CiCMs from the NT group, and those from the 7 days post-mitochondrial delivery group (or 15 days after chemical cardiomyocyte reprogramming), were subjected to hypoxia. This was achieved by incubating the cells at 1% O_2_ concentration for durations of 6 h, 12 h, and 24 h using a hypoxia incubator (#4131, Thermo Fisher Scientific).

*Cell viability*: Cell viability under hypoxic conditions was assessed using a Live/Dead assay. Viability determinations were made at 7 days post-mitochondrial treatment or 15 days subsequent to chemical cardiomyocyte reprogramming. Cells were incubated in a hypoxic chamber for durations of 6 h, 12 h, and 24 h. Subsequently, cells were washed twice with pre-warmed PBS, followed by treatment with a solution containing 2 μM Calcein AM and 4 μM Ethidium homodimer (both purchased from Thermo Fisher Scientific) in PBS. After a 30 min incubation period in the incubator, fluorescence images were captured using an LSM 710 microscope (Carl Zeiss). The quantification of green-labeled live cells and red-stained nuclei of dead cells was conducted using ImageJ software (NIH). In the hypoxia model experiment, XFe 96 plates were coated with 1:50 Matrigel and then seeded with cells at density of 5x10^4^ cells/well. 24 h before Seahorse analysis, the plate was placed in hypoxia incubator to induced hypoxia, following the manufacturer’s instructions.

*In vivo CiCMs treatment*: Crl:CD (SD) rats (190 to 210 g, 7-week-old males; Orientbio) were anesthetized with isoflurane and intubated through the trachea using an 18G intravenous catheter. The rats were ventilated using a rodent respirator (Harvard Apparatus, Holliston, MA, USA). A 37 ℃ heating pad was used to maintain the body temperature throughout the operation. The chest area was shaved and sterilized with 70% alcohol. The animals were divided into two experimental groups (*n* = 3 per group): one receiving untreated CiCMs (1 × 10^6^ cells) and the other receiving mitochondria-treated CiCMs (1 × 10^6^ cells). All groups underwent trypsinization and were labeled with 2 μM CellTracker CM-DiI dye (#C7000, Thermo Fisher Scientific) by incubating the cells for 5 minutes at 37℃. A 60 μl volume of cells was injected at two different sites of left ventricle. The chest was then closed aseptically. The Institutional Animal Care and Use Committee of Yonsei University College of Medicine granted approval for the animal experiments under permit number 2022-0336. All animal experiments were carried out in a facility accredited by the Association for Assessment and Accreditation of Laboratory Animal Care International (AAALAC International).

Supplementary Figures


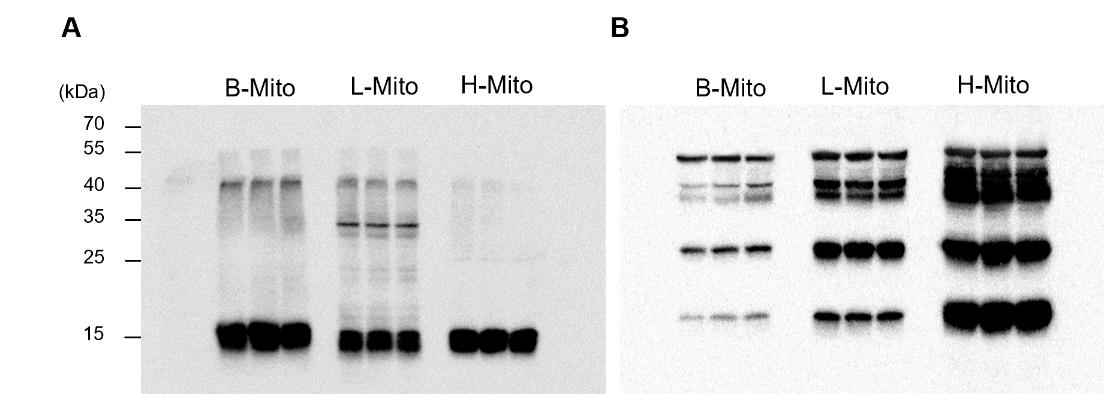


**Figure S1. Original images of western blot data in Figure 1H.** (A) TOM20 expression and (B) mitochondrial OXPHOS complex protein subunits I~V expressions measured in isolated mitochondria samples.; Complex I, NDUFB8 (20 kDa); Complex II, SDHB (30 kDa); Complex III, UQCRC2 (48 kDa); Complex IV, MTCO1 (40 kDa); Complex V, ATP5A (55 kDa).


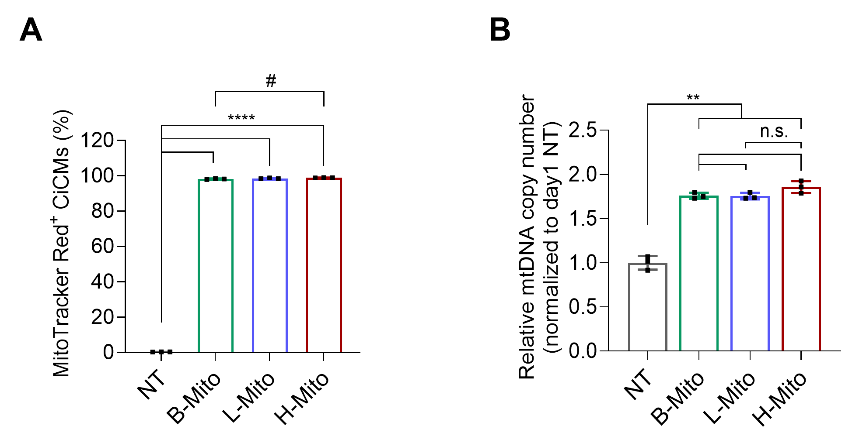


**Figure S2. Quantitative analysis of flow cytometry and real-time PCR results of the mean mtDNA copy number of mitochondria after 24 hours of exogenous mitochondrial delivery.** (A) Transfer efficiency of three types of isolated mitochondria into CiCMs after 24 h post mitochondrial delivery (*n* = 3, B-Mito 98.21 ± 0.32%; L-Mito 98.54 ± 0.24%; H-Mito 98.93 ± 0.09%). All data were calculated with NT group as gating control and expressed as the means ± SD. (B) mtDNA copy numbers relative to NT group levels. Statistical significance between groups was determined using two-way ANOVA followed by Tukey's post hoc test. ***P* < 0.01 and *****P* < 0.0001 versus NT, #*P* < 0.05 versus B-Mito. n.s. indicates no significance.


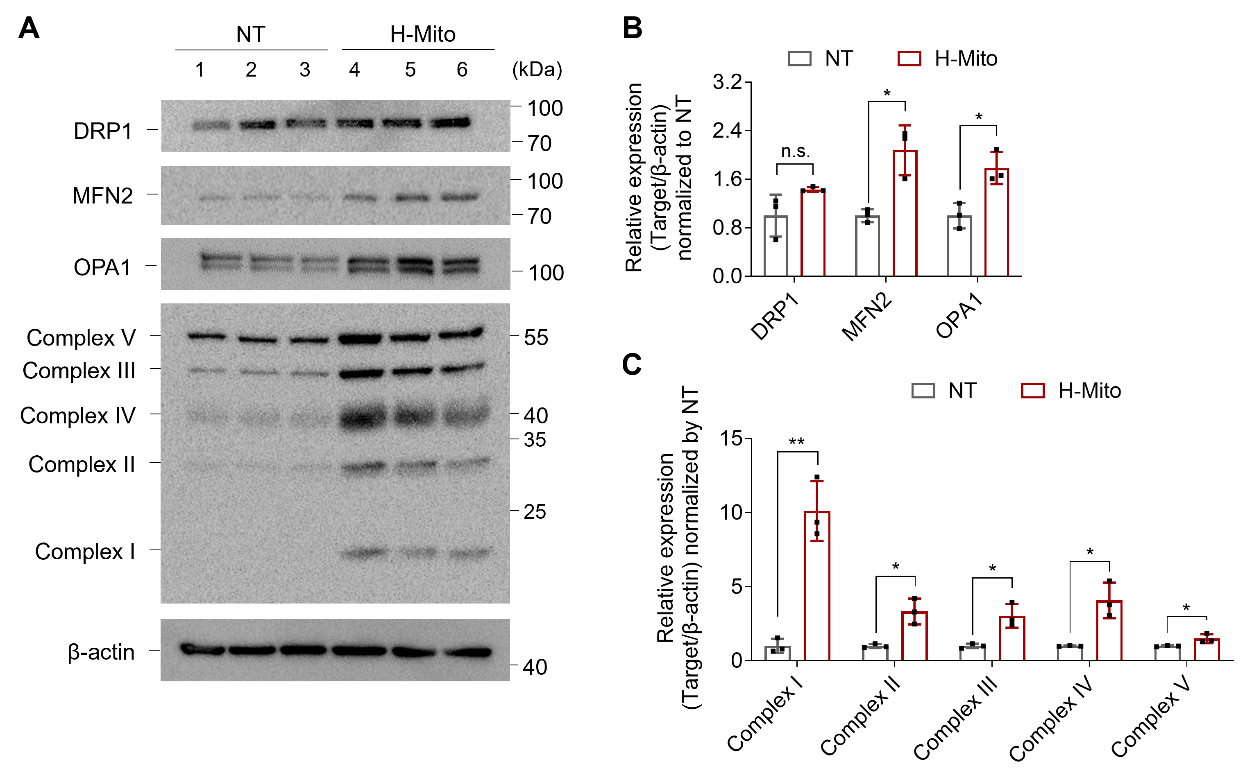


**Figure S3. Western blot analysis of CiCMs after 24 h of heart mitochondria delivery.** (A) Mitochondrial fusion/fission proteins and OXPHOS complex protein subunits I~V expressions were measured with β-actin as a loading control. DRP1 (80kDa); MFN2 (80kDa); OPA1 (80-100kDa); Complex I, NDUFB8 (20 kDa); Complex II, SDHB (30 kDa); Complex III, UQCRC2 (48 kDa); Complex IV, MTCO1 (40 kDa); Complex V, ATP5A (55 kDa); β-actin (45kDa). (B) Relative expression of fusion/fission proteins quantified by densitometry and normalized to that of β-actin (*n* = 3). (C) Relative expression of OXPHOS complex protein subunits quantified by densitometry and normalized to that of β-actin (*n* = 3). All data are expressed as the means ± SD. Statistical significance between groups was determined using two-tailed t test. **P* < 0.05 and ***P* < 0.01 versus NT.

**
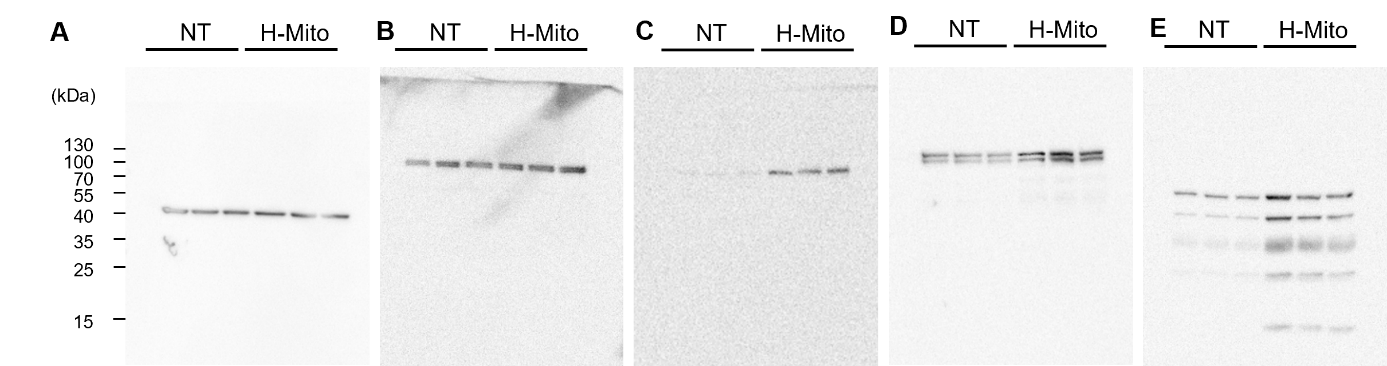
**

**Figure S4. Original images of western blot data in Figure S3A.** (A) β-actin (45kDa), (B) DRP1 (80kDa), (C) MFN2 (80kDa), (D) OPA1 (80-100kDa), and (E) OXPHOS complex protein subunits I~V expressions measured in metabolically reprogrammed CiCMs samples.


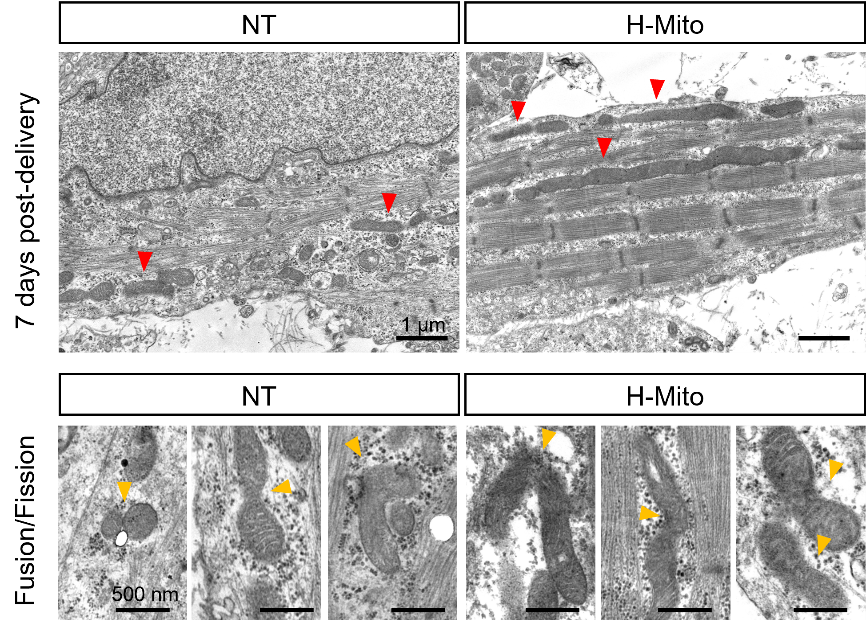


**Figure S5. TEM images displaying CiCMs 7 days post heart mitochondrial treatment (or at 17 days of chemical reprogramming).** Top images, marked with red arrows, indicate elongated mitochondria. Bottom images, highlighted with yellow arrows, depict mitochondria in the midst of fusion/fission events. Scale bars are 1 μm for the top images and 500 nm for the bottom images.


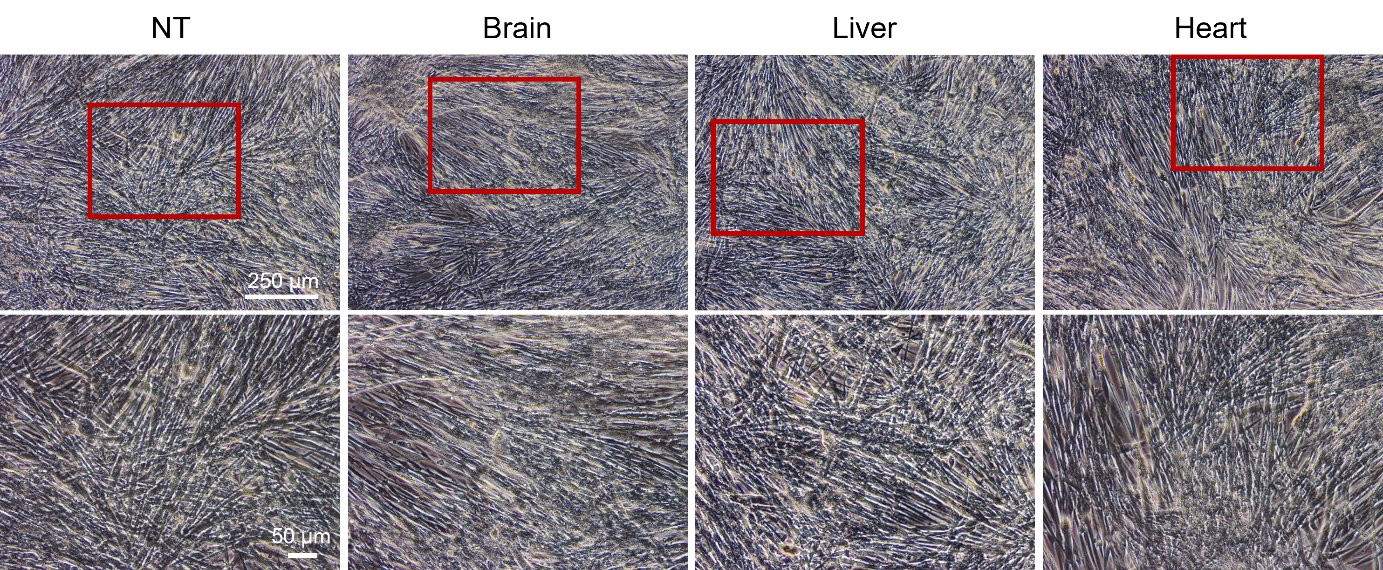


**Figure S6. Bright-field images of CiCMs after 7 days of mitochondrial treatment (or after 17 days of chemical reprogramming).** The red squares indicate the enlarged areas. (Top images scale bar = 200 μm, bottom images scale bar = 50 μm)


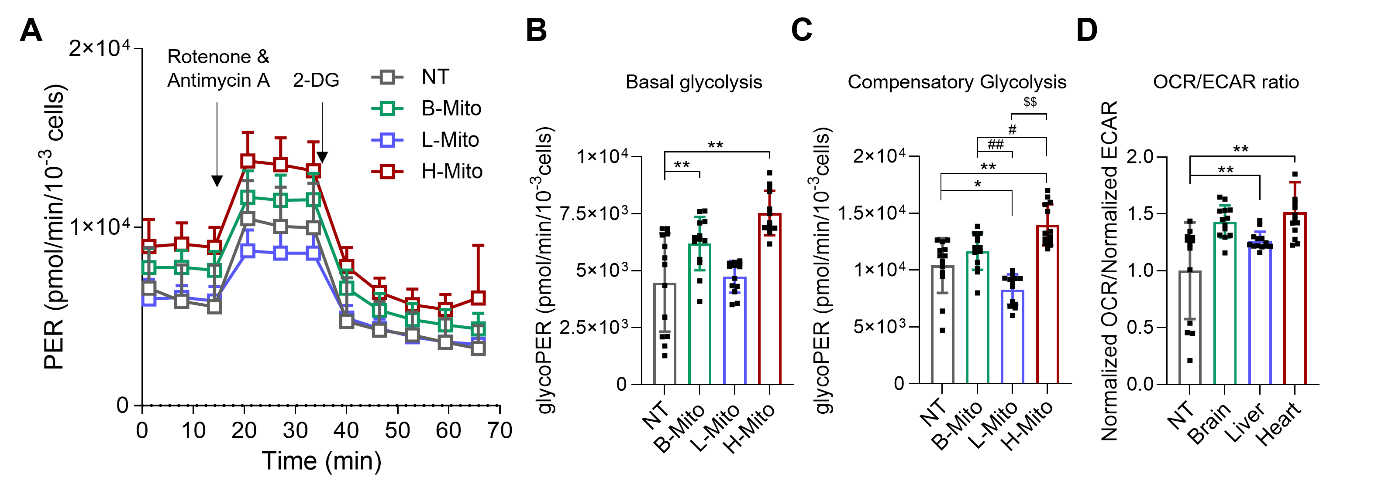
**Figure S7. Glycolytic rate assay after mitochondrial reprogramming of CiCMs.** (A) Proton efflux rate (PER) measurements in untreated CiCMs (NT) and CiCMs treated with mitochondria isolated from Brain (B-Mito), Liver (L-Mito), and Heart (H-Mito) tissues (*n* = 13). The glycolytic rate assay was monitored for 68 minutes. Rotenone and antimycin A, complex I and III inhibitors, were introduced at 16 minutes, followed by the glycolysis inhibitor 2-Deoxy-D-glucose (2-DG) at 36 minutes. (B) Basal glycolysis (*n* = 13). (C) Compensatory glycolysis (*n* = 13). (D) The basal normalized oxygen consumption rate (OCR) to normalized extracellular acidification rate (ECAR) (*n* = 13). Statistical significance between groups was determined using two-way ANOVA followed by Tukey's post hoc test. **P* < 0.05 and ***P* < 0.01 versus NT, #*P* < 0.05 and ##*P* < 0.01 versus B-Mito, $$*P* < 0.01 versus L-Mito group).


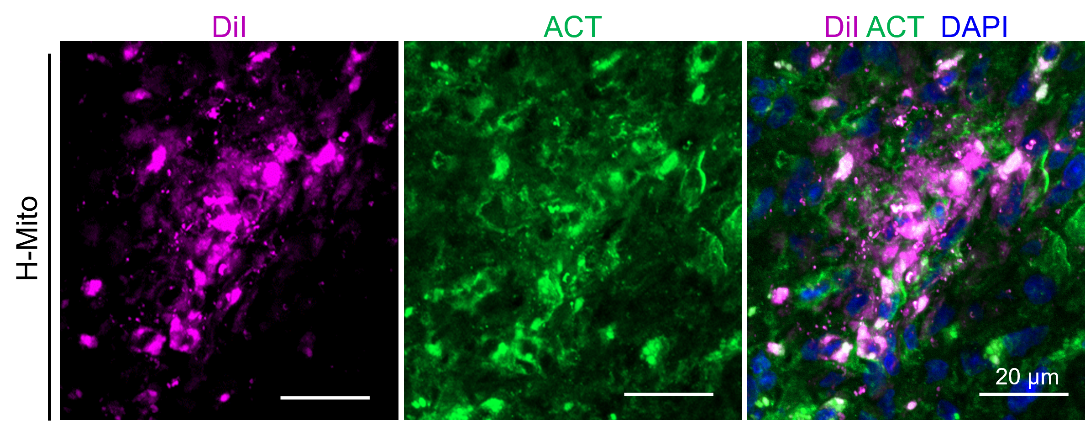


**Figure S8. Immunohistochemical staining of rat heart tissue sections showing alpha-actinin (α-ACT) and DiI-labeled H-Mito CiCMs 7 days post-injection.** The first panel shows DiI-labeled H-Mito CiCMs (magenta), the second panel displays alpha-actinin staining (green), and the third panel combines DiI, ACT, and DAPI (blue) staining to illustrate the integration and survival of H-Mito CiCMs within the heart tissue (scale bars = 20 μm).
